# APKASS 2024 consensus statement on anterior cruciate ligament reconstruction, Part I: Management of paediatric anterior cruciate ligament injury

**DOI:** 10.1016/j.asmart.2026.05.007

**Published:** 2026-06-04

**Authors:** Ming Wang, Yuichi Hoshino, Gilbert Moatshe, Joon Ho Wang, Pakapon Issaragrisil, Stefan Mogos, Nadhaporn Saengpetch, Chanakarn Phornphutkul, Jin Goo Kim, Ryosuke Kuroda, Nattha Kulkamthorn, Hideyuki Koga, Shuhei Otsuki, Ryuichiro Akagi, Sang-Hak Lee, Karl Eriksson, Kyoung Ho Yoon, Bancha Chernchujit, Ekavit Keyurapan, Vasileios Raoulis, Rainer Siebold, Jonathan Patrick Ng, Patrick Shu-Hang Yung, Michael Tim-Yun Ong

**Affiliations:** a Department of Orthopaedics & Traumatology, Faculty of Medicine, The Chinese University of Hong Kong, Hong Kong Special Administrative Region of China; b Department of Orthopaedic Surgery, Kobe University Graduate School of Medicine, Kobe City, Japan; c Oslo Sports Trauma Research Center, Norwegian School of Sport Sciences, Oslo, Norway; d Department of Orthopaedic Surgery, Samsung Medical Center, Sungkyunkwan University School of Medicine, Seoul, Republic of Korea; e Bangkok Academy of Sports and Exercise Medicine (BASEM), FIFA Medical Center of Excellence-FIFA, Bangkok Hospital Headquarters, Bangkok, Thailand; f Nord Hospital, Bucharest, Romania; g Department of Orthopaedics, Faculty of Medicine Ramathibodi Hospital, Mahidol University, Bangkok, Thailand; h Department of Orthopedics, Faculty of Medicine, Chiangmai University, Chiangmai, Thailand; i Department of Orthopedic Surgery and Sports Center, Myong-Ji Hospital, Seoul, Republic of Korea; j Department of Orthopaedics, Phramongkutklao Hospital and College of Medicine, Bangkok, Thailand; k Department of Joint Surgery and Sports Medicine, Graduate School of Medical and Dental Sciences, Institute of Science Tokyo, Tokyo, Japan; l Department of Orthopedic Surgery, Osaka Medical and Pharmaceutical University, Takatsuki, Japan; m Knee Surgery and Sports Medicine Center, Oyumino Central Hospital, Chiba, Chiba, Japan; n Department of Orthopedic Surgery, Center for Joint Diseases and Rheumatism, Kyung Hee University Hospital, Seoul, Republic of Korea; o Department of Orthopaedics, Stockholm South Hospital, Karolinska Institutet, Stockholm, Sweden; p Department of Orthopedic Surgery, Kyung Hee University Hospital, Seoul, Republic of Korea; q Department of Orthopaedics, Faculty of Medicine, Thammasat University, Bangkok, Thailand; r Faculty of Medicine, Siriraj Hospital, Mahidol University, Bangkok, Thailand; s Department of Sports Medicine, University Hospital of Larissa, Larissa, Greece; t International Center for Orthopedics, ATOS Clinic, Heidelberg, Germany

## Abstract

**Background:**

The greater training load and early specialization in youth sports has led to an alarming increase in knee injuries, particularly anterior cruciate ligament (ACL) injuries among children and adolescents. This study aimed to develop a consensus among surgeons and experts from the Asia-Pacific Knee, Arthroscopy and Sports Medicine Society (APKASS) on key aspects of paediatric ACL injury management to enhance clinical outcomes.

**Methods:**

Twenty-three expert surgeons from 8 countries participated in the consensus meeting, which focused on nine crucial domains: prevention, diagnosis, management, surgical techniques, post-operative care, management of associated injuries, rehabilitation, outcome evaluation, and future directions. A predefined agreement threshold of 75% was used to determine consensus. Responses were analysed alongside current literature to pinpoint areas of agreement and divergence.

**Results:**

The consensus project revealed strong agreement on several key aspects of paediatric ACL injury management, particularly the implementation of injury prevention programmes for high-risk sports (85%) and the importance of assessing concomitant meniscal or chondral injuries for surgical intervention (91.3%). However, significant variability was observed in areas such as diagnostic challenges, treatment timing, and rehabilitation protocols. There was no consensus on optimal surgical techniques for skeletally immature patients or the ideal graft diameter for reconstruction. Individualized treatment approaches were emphasized, with 78.3% of surgeons advocating for case-by-case decision-making, reflecting the need to consider factors like skeletal maturity, activity level, and family preferences. There was also a strong consensus on the need for routine monitoring of growth disturbances post-surgery and a comprehensive multi-criteria approach for return-to-sport assessments, underscoring the complexity of managing paediatric ACL injuries.

**Conclusion:**

This expert consensus, developed from an Asia-Pacific perspective, showed both agreement and variation in managing paediatric ACL injuries. While strong consensus was achieved on diagnostic and immediate post-injury care, differences remained in surgical timing, graft selection, and rehabilitation. These findings highlight the need for region specific and paediatric focused guidelines to optimise long term outcomes and establish standardised evaluation criteria for this unique population.

**Level of evidence:**

V (Expert opinion).

## 1 Introduction

The landscape of youth sports has transformed dramatically over the past few decades, characterized by greater training load and early sports specialization.^1^^,^^2^ This change has contributed to a steady rise in knee injuries among children and adolescents, with anterior cruciate ligament (ACL) injuries emerging as a particularly concerning trend.^3^ Global epidemiological data underscores this challenge: A Finnish national registry analysis demonstrated a twofold increase in paediatric ACL injuries between 1997 and 2014.^4^ Similarly, Australian data spanning two decades reveals a substantial increase in knee injuries, with ACL injuries being a majority, particularly among the paediatric population aged 5-18 years.^3^ This trend is also supported by insurance data analysis, which showed a 2.3% annual increase in ACL tears among patients aged 6 to 18.^5^ This increasing injury incidence has been accompanied by a parallel increase in paediatric ACL reconstruction procedures over the past two decades.^6^^,^^7^

The impact of paediatric ACL injuries extends far beyond the immediate trauma. As the primary stabilizer preventing anterior tibial translation, ACL disruption in skeletally immature patients presents unique challenges that distinguish it from adult injuries. The consequences are both immediate and long-term: decreased sports participation, inability to return to play, compromised quality of life, and a higher risk of early-onset osteoarthritis (OA). The open growth plates in paediatric patients further complicates management, as both the injury and its associated surgical intervention can potentially disturb normal growth patterns. Furthermore, considering the longer life expectancy of paediatric patients, the development of post-traumatic OA becomes more concerning, with many patients experiencing knee pain and functional limitations within a decade of injury without timely and appropriate intervention. Beyond these impacts, paediatric ACL injuries also impose substantial economic burdens on healthcare systems and society, including direct healthcare costs and potential long-term disability.

In 2018, the International Olympic Committee convened a global expert group to establish consensus statements on paediatric ACL injuries. This group included orthopaedic surgeons and physiotherapists representing major international organisations: the American Orthopaedic Society for Sports Medicine (AOSSM), European Paediatric Orthopaedic Society (EPOS), European Society for Sports Traumatology, Knee Surgery and Arthroscopy (ESSKA), International Society of Arthroscopy Knee Surgery and Orthopaedic Sports Medicine (ISAKOS), Paediatric Orthopaedic Society of North America (POSNA), and Sociedad Latinoamericana de Artroscopia, Rodilla y Deporte (SLARD). While their comprehensive consensus statement provided valuable support for clinicians, affected children, and parents in decision-making, they notably lacked Asian perspective and experience. This geographical limitation is particularly significant as clinical practices, patient populations, and healthcare delivery systems vary considerably across regions. Despite existing guidelines and consensus, many key topics in paediatric ACL injury management remain under discussion. These include prevention strategies, optimal timing and indications for surgical intervention, selection of appropriate surgical techniques, post-operative rehabilitation protocols, outcome assessment methods, and management of associated injuries. With its distinct healthcare landscape, the Asia-Pacific region presents unique challenges and opportunities in managing paediatric ACL injuries. These regional variations necessitate specific guidelines or consensus that acknowledge and address these features. The Asia-Pacific Knee, Arthroscopy and Sports Medicine Society (APKASS) initiated a consensus meeting to address paediatric ACL injury management in response to these challenges. The meeting focused on nine crucial domains: prevention, diagnosis, management, surgical techniques, post-operative care, management of associated injuries, rehabilitation, outcome evaluation, and future directions. This consensus statement seeks to bridge existing knowledge gaps by integrating evidence-based practices with regional expertise, systematically synthesizing expert opinion from the Asia-Pacific region.

## 2 Methods

The Asia-Pacific Knee, Arthroscopy and Sports Medicine Society (APKASS) established an Asia-Pacific ACL consensus project in October 2024, focusing on paediatric ACL injury management. The expert panel comprised 23 experienced orthopaedic surgeons specializing in knee sports injuries, including 18 APKASS fellows and 5 European Society of Sports Traumatology, Knee Surgery and Arthroscopy (ESSKA) fellows.

**Fig. 1:**
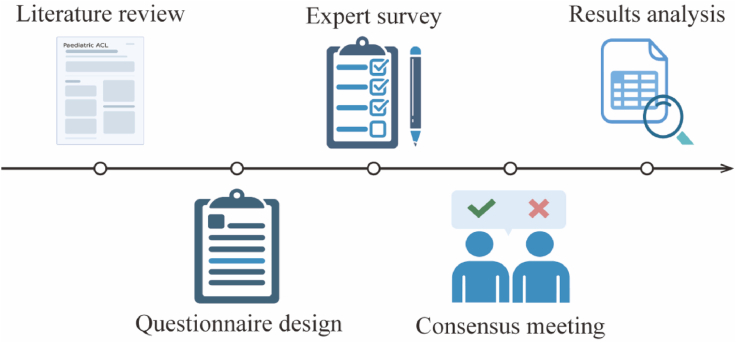
The flowchart of the study.

The steering committee consisted of three post-doctoral research fellows (MW, ZL, MC), two expert knee surgeons (JPN, MTYO), and one lead specialist in ACL injury management (PSHY). This committee was responsible for developing survey questions and conducting a comprehensive literature review. A literature search was performed using PubMed and Embase databases with combinations of relevant search terms, including "anterior cruciate ligament," "injury," "rupture," "reconstruction," "arthroscopy," "arthroscopic," "paediatric," "paediatric," "child," "children," and "adolescent." Animal and cadaver studies were excluded from the review. Based on the literature review, the steering committee developed 28 questions across nine domains: prevention, epidemiology, diagnosis, treatment, surgical techniques, rehabilitation, outcome measures, return to sport, and future research priorities. Hard copies of the survey were distributed to all 23 panel members during the consensus meeting. The consensus meeting was held during the 2024 APKASS Congress on October 24, 2024. During this meeting, participants completed the survey questionnaires, and results were collected and discussed, with particular attention to identifying areas of agreement and differing practices among experts (See Fig. 1). We assess consensus using percentage agreement. A statement or item must meet the predetermined threshold of 75% in order to be said to have achieved consensus. The primary objective was to evaluate current management approaches, document variations in clinical practice, and identify areas requiring further research in paediatric ACL injury management.

Survey responses were analysed using descriptive statistics to characterize current practices and perspectives among participating surgeons. This methodological approach provided insights into the diversity of management strategies and highlighted areas where consensus exists and where further evidence is needed to guide clinical practice.

## 3 Discussion

Following a thorough, deliberative process, a definitive consensus was achieved on 16 questions related to the management of paediatric ACL injuries. As summarized in Table 1, these results reflect a expert opinion on several critical topics. The following sections will provide a detailed discussion of the findings for each area of consensus, offering insights into current best practices and future directions.

**Table 1:** APKASS paediatric ACL injury consensus.

APKASS Paediatric ACL injury Consensus
1. The expert panel recommends early implementation of injury prevention programs for children and adolescents participating in high-risk sports.
2. The consensus suggests that the management approach for paediatric ACL injuries be determined on a case-by-case basis, tailored to the individual patient.
3. **The expert panel recommends that** the presence of concomitant meniscal or chondral injuries be considered a critical factor necessitating surgical intervention.
4. **The expert panel recommends that** transphyseal ACL reconstruction be the surgical technique of choice for older adolescents nearing skeletal maturity.
**5. The expert panel recommends that** unstable meniscal tears in adolescents be repaired concurrently with ACL reconstruction to preserve long-term knee function.
**6. The expert panel recommends that** hamstring autograft be the preferred choice for all-epiphyseal ACL reconstruction in paediatric patients.
**7. The expert panel recommends that** the use of bone-patellar tendon-bone (BTB) autografts be avoided in paediatric patients with open growth plates due to the significant risk of growth disturbance.
**8. The expert panel recommends that** allografts not be used for paediatric ACL reconstruction.
**9. The expert panel recommends that** single-bundle ACL reconstruction be performed for skeletally immature patients.
10. **The expert panel recommends that** growth disturbances be routinely monitored after paediatric ACL reconstruction to facilitate early detection and intervention.
**11. The expert panel recommends that** meniscal repair be performed in paediatric ACL injuries to preserve knee function and prevent long-term joint degeneration.
**12. The expert panel recommends that** comprehensive neuromuscular training and bilateral rehabilitation be implemented to prevent contralateral ACL injuries.
**13. The expert panel recommends that** readiness for return to sports be assessed using a combination of objective strength testing, functional performance, and time from injury.
**14. The expert panel recommends that** a comprehensive, multi-dimensional evaluation be used to assess outcomes following paediatric ACL reconstruction.
**15. The expert panel recommends that** the top three priorities for future research be prevention strategies, surgical techniques, and biological augmentation.
**16. The expert panel recommends that** current guidelines for managing paediatric ACL injuries be considered insufficient, and that more comprehensive, evidence-based standards be developed.

## 4 Section 1: Prevention of ACL injuries in the paediatric population

### 4.1 Implementation of injury prevention programmes

There is strong consensus (85%) among experts supporting the early implementation of injury prevention programmes for children and adolescents participating in high-risk, pivoting sports. While no single age threshold was defined by the consensus group, the available evidence suggests that injury prevention efforts should begin as early as possible once children engage in these sports. Injury rates have been shown to increase between 10 and 12 years of age, and structured programmes such as FIFA 11+ KIDS, designed for children aged 7 to 13 years, have demonstrated efficacy in reducing sports related injuries in this population.^8^ The FIFA 11+ Kids programmes emphasizes cognitive awareness (spatial orientation and anticipation), general movement coordination, and safe falling techniques to prevent injuries in young players.^9^ Multiple systematic reviews and meta-analyses have shown that structured prevention programmes can reduce the overall risk of injuries around 50% when properly implemented.^9^ The challenge of consistent programmes adherence remains a significant consideration, as effectiveness is directly correlated with regular participation and proper execution of the prescribed exercises.^10^

## 5 Section 2: Diagnosis of ACL injuries in Paediatric Patients

### 5.1 Challenges in paediatric ACL injury diagnosis

No consensus was reached regarding the primary challenge in diagnosing paediatric ACL injuries, reflecting the complexity of the process. The accurate diagnosis of paediatric ACL injuries presents challenges for surgeons. Communication with patients and families emerged as the most frequently reported challenge (56.5% of surgeons), as it is critical for understanding symptoms and guiding treatment decisions.^11^^,^^12^ Additionally, 43.5% of surgeons found interpreting imaging difficult, largely due to developmental variations in the growing skeleton.^13^ Physical examination is also complicated by greater joint laxity in children (39.1%), and obtaining accurate injury histories can be challenging given the young age of patients (34.8%). These findings highlight the numerous challenges involved in paediatric ACL in diagnosis and emphasize the need for an integrated approach that combines thorough clinical assessment, careful imaging interpretation, and clear communication with both patients and their families.

## 6 Section 3: Treatment of ACL injuries in Paediatric Patients

### 6.1 Treatment for paediatric ACL injuries

Consensus was reached (78.3%) that treatment decisions for paediatric ACL injuries should be made on a case-by-case basis. The individualized approach to treating paediatric ACL injuries reflects the complexity and variability of the cases. Multiple studies support this strategy, as treatment decisions must account for various factors, including skeletal maturity, physical activity level, concurrent injuries, surgery-associated risks, and family preferences. The high consensus for individualized decision-making aligns with current evidence.^12^ While some surgeons favor early surgical intervention (13%) to prevent secondary injuries and restore knee stability, the case-by-case approach allows for consideration of crucial factors such as growth plate status, sport-specific demands, and family compliance with treatment protocols.

### 6.2 Factors influencing ACL surgery vs. non-operative decisions

No single factor reached consensus in the decision between surgical and non-operative management of paediatric ACL injuries. Surgeons highlighted multiple considerations of comparable importance: associated injuries (73.9%), activity level (69.6%), skeletal maturity (60.9%), and patient/family preference (60.9%). Studies have shown that associated injuries, particularly meniscal tears,^14^ can significantly influence the timing and necessity of long-term outcomes.^15^^,^^16^ The equal weighting of skeletal maturity and family preferences reflects the delicate balance between biological and psychosocial factors in paediatric ACL injuries.

### 6.3 Management of adolescent stable ACL injuries

No consensus was reached on optimal management for adolescent athletes in non-pivoting sports with stable knees after physical therapy. Studies have demonstrated variable outcomes with both operative^16^^,^^17^ and non-operative^18^ management in this paediatric population. The variation in treatment preferences likely reflects the complex interplay between patient factors, long-term outcomes, and the evolving evidence in paediatric sports medicine.

### 6.4 First-line management: considering secondary injury risk

No consensus was reached on the initial management of paediatric ACL injuries. The majority of surgeons (60.9%) favored beginning with conservative measures while considering early surgery in selected cases, whereas 17.4% recommended conservative treatment alone and 21.7% preferred early surgical intervention. This variation reflects the ongoing debate regarding the balance between delaying surgery to allow for psychological and skeletal maturity, thereby improving compliance and reducing growth-related risks, versus the potential benefits of early reconstruction in reducing secondary meniscal and chondral injuries.^19^

### 6.5 Factors influencing surgical recommendation

Consensus was reached (91.3%) that concomitant meniscal or chondral injuries are the most critical factor influencing surgical management of skeletally immature ACL tears. This agreement reflects strong evidence showing that untreated concomitant injuries accelerate joint degeneration and worsen long-term outcomes. Other factors included persistent instability after rehabilitation (73.9%) and the desire to return to competitive pivoting sports (69.6%), though neither reached consensus. The emphasis on associated injuries is consistent with current literature, supporting early surgical intervention in the presence of meniscal or chondral pathology to prevent progressive joint damage.^12^

### 6.6 Methods to assess skeletal maturity

No consensus was reached on the preferred method of assessing skeletal maturity in paediatric ACL injury evaluation. Physiologic age assessment (e.g., Tanner staging) was most frequently selected (73.9%), followed by skeletal age determination (65.2%) and MRI evaluation (52.2%), while chronological age was less commonly used (26.1%). This variability highlights the recognition that no single method is sufficient for accurately determining growth potential. Current evidence supports combining multiple assessment tools to improve reliability and guide safe surgical planning.^12^

### 6.7 Recommended ACL reconstruction technique for paediatric patients

ACL reconstruction technique selection in paediatric patients shows a clear age-dependent pattern, with consensus reached only in the oldest adolescent group. Strong consensus was reached for transphyseal reconstruction. At this stage, surgical techniques closely resemble adult approaches, with reduced concern for physeal injury and greater surgeon familiarity with standard procedures. This result shows the balance between protecting the growth plate in younger children and ensuring surgical efficacy in more mature adolescents. The variability in early adolescence highlights the importance of individualized assessment of skeletal maturity and growth potential.^20^^,^^21^ The transphyseal technique in the older adolescents is similar to the technique for adults because the surgeon is more likely to be familiar with the procedure, and it may reduce the risk of complications.^12^

### 6.8 Timing of ACL reconstruction

No consensus was reached on the optimal timing of ACL reconstruction in paediatric patients. The most frequently selected approach was extended rehabilitation for 6–12 weeks (34.8%), followed by a short delay of 3–6 weeks (21.7%) or delaying surgery until skeletal maturity (21.7%). Immediate surgery within 3 weeks (8.7%) was less common. Two surgeons (8.7%) reported different approaches, incorporating stability and associated injuries to guide timing.

This variability reflects the complexity of balancing early reconstruction to reduce secondary meniscal injury risk against potential benefits of delaying surgery to allow further growth and psychological readiness. Notably, a recent meta-analysis demonstrated that delaying ACL reconstruction beyond 12 weeks significantly increases the risk of meniscal injuries and irreparable meniscal tears.^19^^,^^22^

### 6.9 Management of unstable meniscal tears

Complete consensus was reached (100%) that unstable meniscal tears in adolescents should be repaired simultaneously with ACL reconstruction. This unanimous agreement reflects strong evidence showing superior healing rates and improved long-term outcomes when meniscal repair is performed concurrently with ACL reconstruction, compared to isolated repair^23^^,^^24^ The biological environment created during ACL reconstruction, including increased vascularity and growth factors, has been shown to enhance meniscal healing potential.^25^

### 6.10 Use of lateral extra-articular tenodesis (LET)

No consensus was reached regarding the use of lateral extra-articular tenodesis (LET) in adolescent ACL reconstruction. The most common approach was selective use in high-risk cases (43.5%), while others reported rarely performing LET (26.1%), never using it (21.7%), or routinely incorporating it (8.7%). Emerging evidence suggests that adding LET to ACL reconstruction in adolescents may reduce graft rupture rates without increasing non-graft-related reoperations or complications.^26^ The role of LET in adolescent ACL reconstruction remains controversial. Further high-quality prospective studies are needed to clarify its indications and long-term outcomes.

## 7 Section 4: Graft choice and surgical techniques

### 7.1 Preferred graft for all-epiphyseal ACL reconstruction

Strong consensus was reached (91.3%) in favor of hamstring autograft as the preferred graft for all-epiphyseal ACL reconstruction in paediatric patients. Only 8.7% of surgeons indicated that graft choice depends on individual patient factors, while no respondents selected quadriceps tendon, bone-patellar tendon-bone (BTB) autograft, or allograft. This consensus aligns with current evidence and technical considerations. The all-epiphyseal technique avoids physeal violation by confining bone tunnels to the distal femoral and proximal tibial epiphysis.^27^ However, a systematic review by Longo et al. reported that the overall rate of growth disturbance after ACL reconstruction in skeletally immature patients was 2.6%, with no statistical difference between transphyseal and physeal sparing techniques, suggesting that the all-epiphyseal technique may not completely eliminate physeal injury risk.^28^ Notably, current clinical evidence supports the use of soft tissue grafts in skeletally immature patients.^29^, ^30^, ^31^.

### 7.2 BTB autograft in paediatric patients with open physes

Strong consensus was reached (82.6%) that bone-patellar tendon-bone (BTB) autografts should be avoided in patients with open physes. The remaining surgeons reported not using BTB autografts at all in their practice (17.4%). This consensus reflects concerns regarding physeal damage and growth-related complications associated with BTB autografts.^31^ While their use may be considered in patients nearing skeletal maturity with nearly closed physes, they are generally contraindicated in skeletally immature patients with more than one year of growth remaining.^12^^,^^31^ This cautious approach is warranted due to two primary risks: potential deleterious effects from BTB graft harvest itself and the risks associated with placing bone plugs across open physes.

### 7.3 Use of allografts for ACL reconstruction

Strong consensus was reached against the use of allografts for adolescent ACL reconstruction. Only 17.4% (4/23) surgeons reported selective use, typically reserved for revision surgeries or multi-ligament injuries. This consensus against allografts is consistent with current literature, which demonstrates inferior outcomes and higher graft failure rates in young, active patients.^32^, ^33^, ^34^.

### 7.4 Graft diameter for transphyseal ACL reconstruction

No consensus was reached regarding the optimal graft diameter for transphyseal ACL reconstruction in adolescents. The most common target was 7–8 mm (30.4%), followed by > 8 mm (17.4%) and 6–7 mm (13%). Notably, 21.7% reported no fixed target, tailoring graft size to individual patient factors, while 13% selected “Other,” citing approaches such as knee-dimension–based sizing, avoidance of transphyseal techniques, or use of double-bundle reconstructions with grafts <6 mm each.

This heterogeneity reflects ongoing debate about minimum safe graft size in the paediatric population. While adult literature suggests that grafts <8 mm carry a significantly higher risk of failure, its direct applicability to children remains uncertain.^35^^,^^36^ Patient-specific factors—including weight <50 kg, height <140 cm, thigh circumference <37 cm, and BMI <18—are associated with smaller hamstring graft diameters (<7 mm).^37^^,^^38^ The long-term outcomes of smaller grafts is also unclear: some studies suggest stability or neogenesis in graft over time,^37^ while others report shrinkage and higher rupture risk.^39^

### 7.5 ACL reconstruction technique (double-bundle vs single-bundle)

A consensus was reached in favor of single-bundle ACL reconstruction for skeletally immature patients. Most surgeons exclusively perform single-bundle reconstruction, while 4.3% usually perform single-bundle but consider double-bundle in select cases. A minority reported not performing ACL reconstructions in skeletally immature patients. Notably, no respondents reported routinely using a double-bundle technique. The optimal surgical approach for skeletally immature patients remains a subject of debate. In adults, double-bundle reconstruction more closely restores native ACL anatomy and has been shown to improve rotational stability. However, in skeletally immature patients, the primary concern is growth plate preservation. Despite these potential advantages, the strong preference for single-bundle reconstruction likely reflects practical considerations: technical simplicity, reduced physeal disruption, and equivalent functional outcomes compared to double-bundle techniques.

## 8 Section 5: Post-operative care and risk management

### 8.1 Growth disturbance monitoring after paediatric ACL reconstruction

Strong consensus was reached regarding the importance of routine monitoring for growth disturbances following paediatric ACL reconstruction. This consensus reflects broad recognition of the potential for growth disturbances, including leg length discrepancies and angular deformities, after transphyseal ACL reconstruction. Routine follow-ups and monitoring enables earlier detection and timely intervention, mitigating long-term complications. The variation in intensity of monitoring suggests that most surgeons adapt their protocols based on patient-specific factors such as skeletal maturity, surgical technique, and perceived risk profile.

## 9 Section 6: associated injuries and complications

### 9.1 Management of concomitant meniscal tears

Strong consensus was reached in favor of meniscal preservation in paediatric ACL injuries. Most surgeons (65.2%, 15/23) reported attempting meniscal repair whenever possible, regardless of tear pattern, while the remaining 34.8% (8/23) selectively repair based on tear pattern and location. Meniscal injury is a common finding in paediatric ACL injuries, occurring in approximately 40–60% of cases.^40^^,^^41^ Given the meniscus's critical role in maintaining knee biomechanics, stability, and long-term joint health, meniscal preservation is now prioritized. Studies of revision ACL reconstruction have shown that prior meniscectomy is associated with higher rates of chondral lesions, whereas meniscal repair is not.^42^

### 9.2 Prevention strategies for contralateral ACL injury

Strong consensus was reached in the use of a comprehensive, multi-modal approach to prevent contralateral ACL injury in paediatric patients following ACL reconstruction. The most frequently employed strategies included movement education, bilateral rehabilitation protocols, functional testing to guide return-to-sport decisions, and neuromuscular training. This consensus reflects recognition of the high risk of contralateral ACL injury in young patients, with studies reporting contralateral tear rates as high as 30% within two years post-reconstruction, accounting for up to 70% of all re-injuries.^43^

## 10 Section 7: rehabilitation and return to sport

### 10.1 ACL rehabilitation protocols for paediatric patients

No consensus was reached regarding rehabilitation protocols for paediatric ACL reconstruction. The most common approach was the use of modified adult protocols with adjustments for children (52.2%, 12/23). This distribution suggests that most current approaches are derived from adult rehabilitation protocols, with varying degrees of adaptation for paediatric patients. The relatively limited adoption of paediatric-specific protocols highlights a gap in standardized, evidence-based rehabilitation strategies designed for children. Given the unique physiological, developmental, and psychosocial needs of paediatric patients, the lack of guidelines highlight the importance of developing validated, age-appropriate rehabilitation frameworks.

### 10.2 Return to sports criteria after paediatric ACL reconstruction

Strong consensus was reached regarding the use of multi-factorial criteria to determine return-to-sport readiness in paediatric patients following ACL reconstruction. The most commonly applied measures were strength testing, functional testing, and time from surgery. Psychological readiness, including fear of re injury, confidence in knee function, and emotional preparedness, is also an important factor in return to sport decision making for paediatric and adolescent athletes, as fear of re injury has been shown to be a significant predictor of delayed return to sport and lower performance levels.^44^ Despite widespread use of criteria such as the limb symmetry index (≥90%), studies indicate that only 13–25% of paediatric patients meet all return-to-sport benchmarks after ACL reconstruction.^45^ This aligns with the International Olympic Committee's recommendation to delay return to pivoting sports for at least 12 months post-surgery in youth athletes.^12^

## 11 Section 8: Outcomes evaluation

### 11.1 Outcome measures after paediatric ACL reconstruction

Consensus was reached in favor of using a comprehensive, multi-dimensional evaluation to assess outcomes following paediatric ACL reconstruction. The most frequently utilized measures were knee laxity assessments patient-reported outcome measures (PROMs), re-injury rates, and functional testing 78.3%. This adoption of both objective and subjective measures reflects recognition that surgical success in paediatric ACL reconstruction cannot be assessed simply by single standards. Instead, it requires a systematic approach that incorporates biomechanical parameters, functional recovery, patient experience, and complication monitoring.

### 11.2 Patient reported outcome measures (PROMs) in paediatric ACL reconstruction

No consensus was reached in the PROMs for paediatric ACL injuries. Although PROMs are crucial for understanding a patient's perceived recovery, the current approach lacks standardization. The results shows that most clinicians use adult ACL PROMs without any modifications (47.8%), with fewer using modified adult PROMs (21.7%) or paediatric-specific ones (17.4%). This is concerning because commonly used tools like the Lysholm, IKDC, Tegner Activity Scale, and KOOS were not designed for or validated in a paediatric population. This reliance on adult outcome measures highlights a gap in accurate evaluation of outcomes for paediatric patients and the need to develop and validate appropriate paediatric-specific PROMs.^46^

## 12 Section 9. Future Directions

### 12.1 Future research priorities in paediatric ACL injuries

There is a clear consensus among experts on the most critical areas for future research in paediatric ACL injuries. The top three research fields identified are prevention strategies, surgical techniques, and biological augmentation. This expert consensus emphasizes the importance of preventing injuries, refining surgical methods for better clinical outcomes in skeletally immature patients, and exploring advanced biological therapies to enhance healing.

### 12.2 Current paediatric ACL injury guidelines

A clear consensus is achieved that the current guidelines for managing paediatric ACL injuries are insufficient, with a combined 95.6% of respondents calling for updates. This highlights a need for further discussion and research to establish a comprehensive, widely accepted set of guidelines for the management of paediatric ACL injuries.

## 13 Asia-Pacific perspectives on paediatric ACL management

Several region specific aspects of the Asia-Pacific context warrant emphasis. Access to specialised paediatric sports medicine services varies considerably across the region, from advanced urban centres to resource limited rural areas. Cultural attitudes towards youth sports, with academic achievement often prioritized over athletics in some Asian societies, may influence family preferences for non-operative management. The types of high risk pivoting sports also differ by region, including soccer, basketball, martial arts, rugby, and netball. Additionally, healthcare funding models, ranging from universal coverage to out of pocket payment systems, affect access to ACL reconstruction, graft choices, and rehabilitation. These regional variations highlight the importance of tailoring consensus recommendations to local contexts.

## 14 Limitations

Several methodological limitations of this consensus project should be acknowledged. First, the consensus was achieved through a single round, face to face voting process rather than an anonymous, iterative approach such as the Delphi method. While the face-to-face format facilitated real time discussion and clarification among experts, it may have allowed group dynamics, including the influence of dominant opinions or social conformity, to affect individual responses. In contrast, Delphi based methods use anonymous, sequential survey rounds to progressively refine expert agreement while minimising interpersonal bias. Second, the consensus threshold was set at 75%, which, while consistent with many consensus studies, is arbitrary and may have excluded some clinically relevant viewpoints. Third, the expert panel, although diverse and internationally representative, consisted primarily of surgeons from the Asia-Pacific region, which may limit the generalisability of findings to other geographic or healthcare settings. Future studies employing Delphi methodology with broader geographic representation and formal evidence grading would help validate and extend these findings.

## 15 Conclusion

This consensus project involving 23 experienced knee surgeons from the Asia-Pacific region and Europe revealed both areas of agreement and considerable variation in the management of paediatric ACL injuries. Strong consensus was reached on several key aspects, particularly regarding diagnostic approaches and immediate post-injury management. However, significant variations in practice were observed in surgical timing, graft choice, and return-to-sport protocols. The findings highlight the complexity of managing paediatric ACL injuries and demonstrate that multiple factors, including skeletal maturity, activity level, and family preferences, often influence treatment decisions. This project identifies several areas where further high-quality research is needed, particularly regarding the long-term outcomes of different surgical techniques and optimal rehabilitation protocols in the paediatric population. The results of this consensus project can serve as a framework for clinical decision-making while acknowledging the need for individualized treatment approaches in paediatric ACL injury management.
