# Supplementary material for: APKASS 2024 consensus statement on anterior cruciate ligament reconstruction, Part I: Management of paediatric anterior cruciate ligament injury
Source: Asia Pac J Sports Med Arthrosc Rehabil Technol. 2026 Jun 4;45:26–32. doi: 10.1016/j.asmart.2026.05.007 (PMC13254715; doi:10.1016/j.asmart.2026.05.007)

***Section 1: Prevention of ACL Injuries in paediatric population***

*1. How do you feel about injury prevention programs for kids and adolescents in sports, like FIFA '11+ For Kids'?*

*□ A. Strongly support for all kids in pivoting sports*

*□ B. Recommend for high-risk sports or kids with past injuries*

*□ C. Think they're good but hard to do consistently*

*□ D. Neutral due to limited long-term evidence for kids*

*□ E. Don't usually recommend them*

*□ F. Other (please specify): _______________*

***
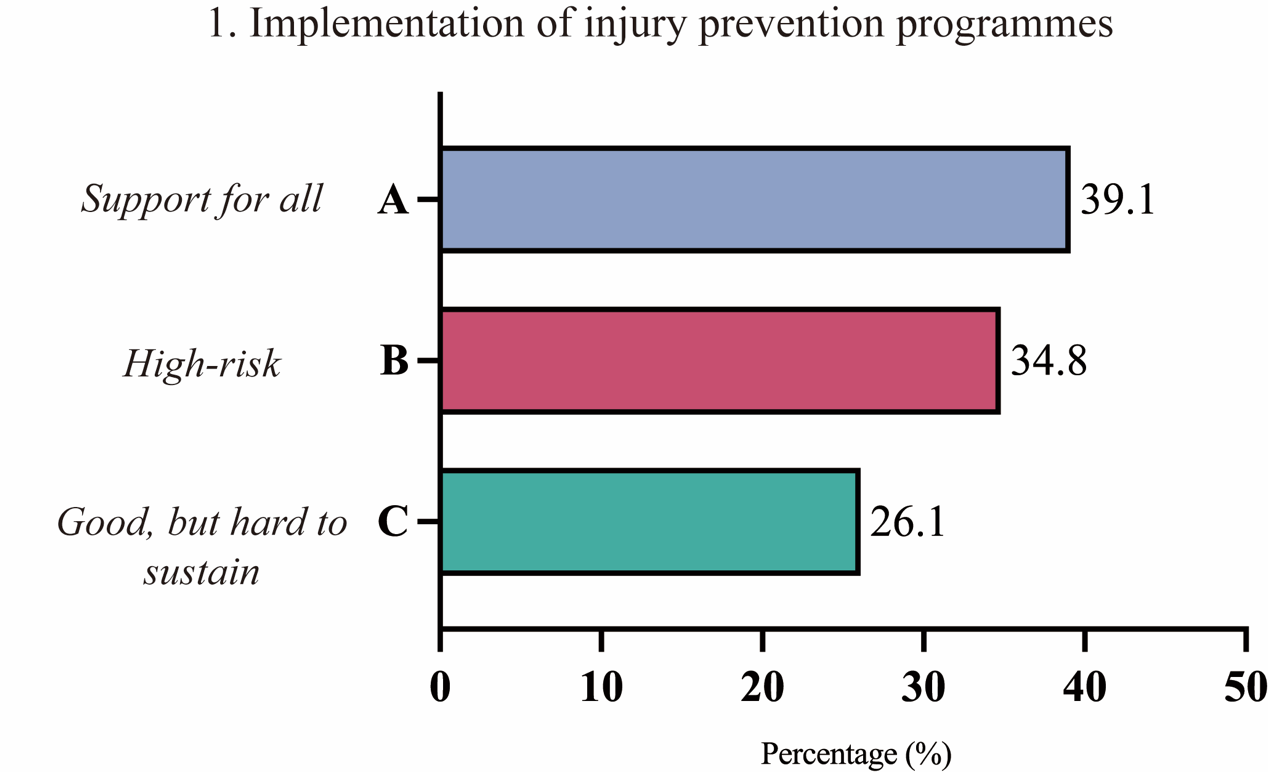
***

***Section 2: Diagnosis of ACL Injuries in Paediatric Patients***

***2. Which challenges do you face when diagnosing ACL injuries in kids? (Tick all that apply)***

*□ A. Physical examination due to greater joint laxity*

*□ B. Difficulty getting accurate history from kids*

*□ C. Interpreting imaging considering developmental variations*

*□ D. Differentiating ACL injuries from other knee pathologies*

*□ E. Choosing the right tests for different ages*

*□ F. Communicating with patients and their parents/guardians*

*□ G. Other (please specify): _______________*


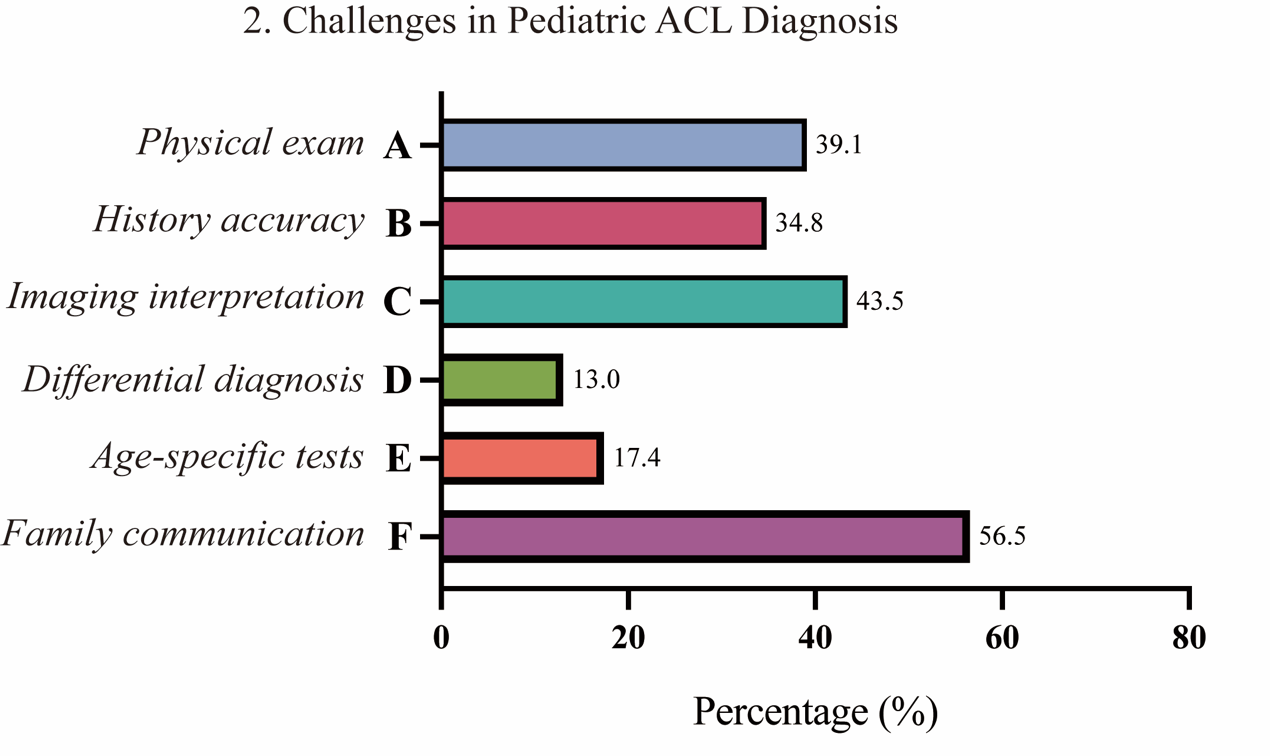


***Section 3: Treatment of ACL Injuries in Paediatric Patients***

***Nonoperative vs. Operative Treatment***

***3. How do you typically approach treatment decisions for ACL injuries in paediatric athletes?***

*□ A. I usually recommend surgery for most cases*

*□ B. I tend to favor non-operative treatment when possible*

*□ C. I make decisions case-by-case based on multiple factors*

*□ D. I follow specific age-based protocols*

*□ E. Other (please specify): _______________*

***
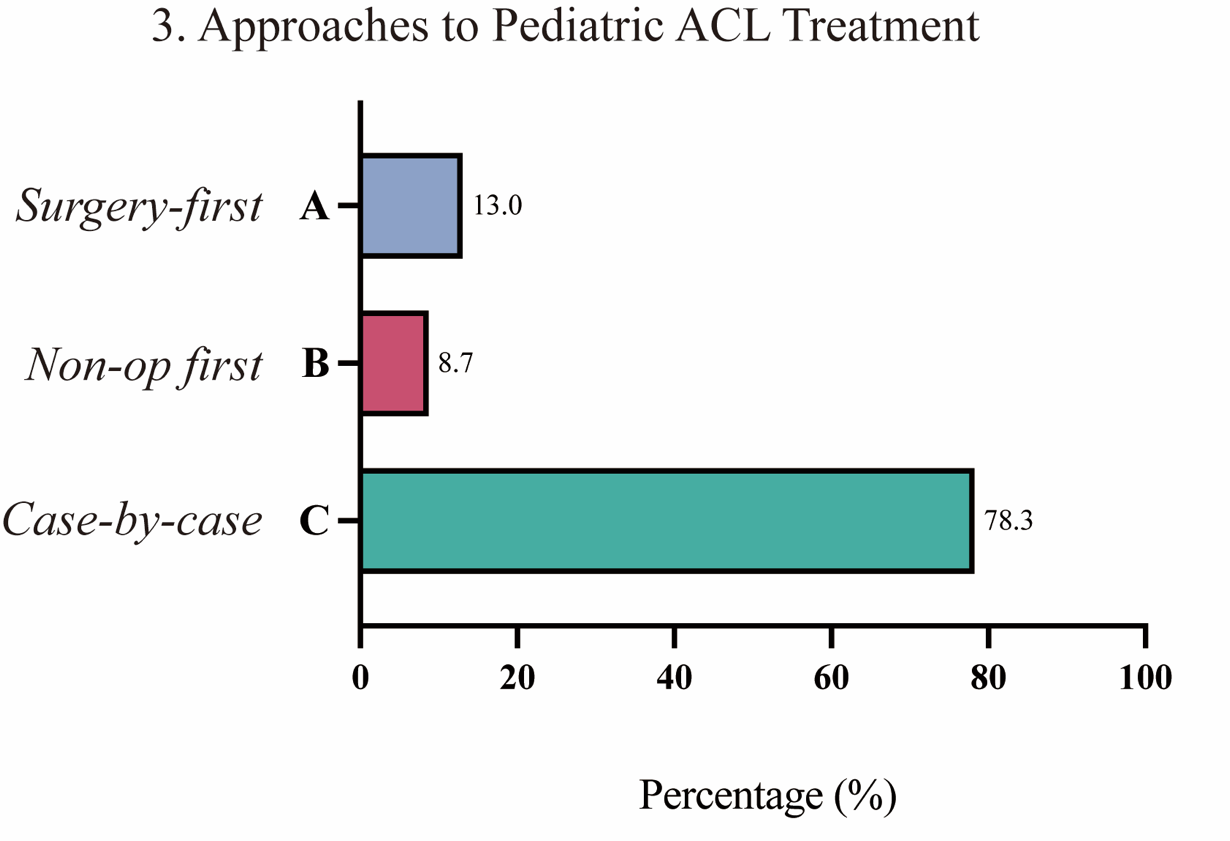
***

***4. What is the most important factor in your decision to perform ACL reconstruction versus non-surgical management in paediatric patients? (Check all that apply)***

*□ A. Skeletal maturity*

*□ B. Activity level*

*□ C. Associated injuries*

*□ D. Patient/family preference*

***
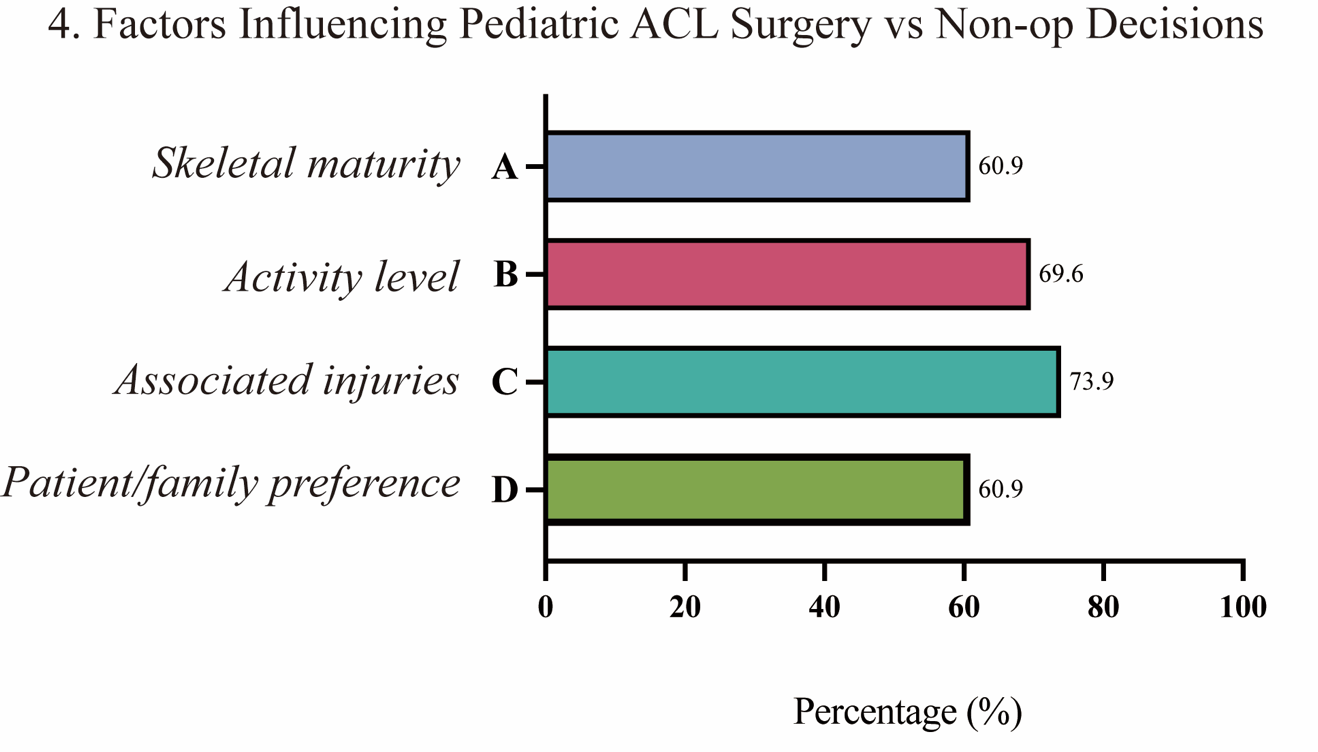
****□ E. Other (please specify): _______________*

***5. How do you typically approach ACL injuries in adolescent patients who don't participate in pivoting sports and show no residual instability after physical therapy?***

*□ A. I almost always recommend non-operative management*

*□ B. I often recommend non-operative management but consider surgery in some cases*

*□ C. I usually recommend surgery but consider nonoperative management in some cases*

*□ D. I almost always recommend surgery regardless of activity level or stability*

*□ E. Other (please specify): _______________*

***
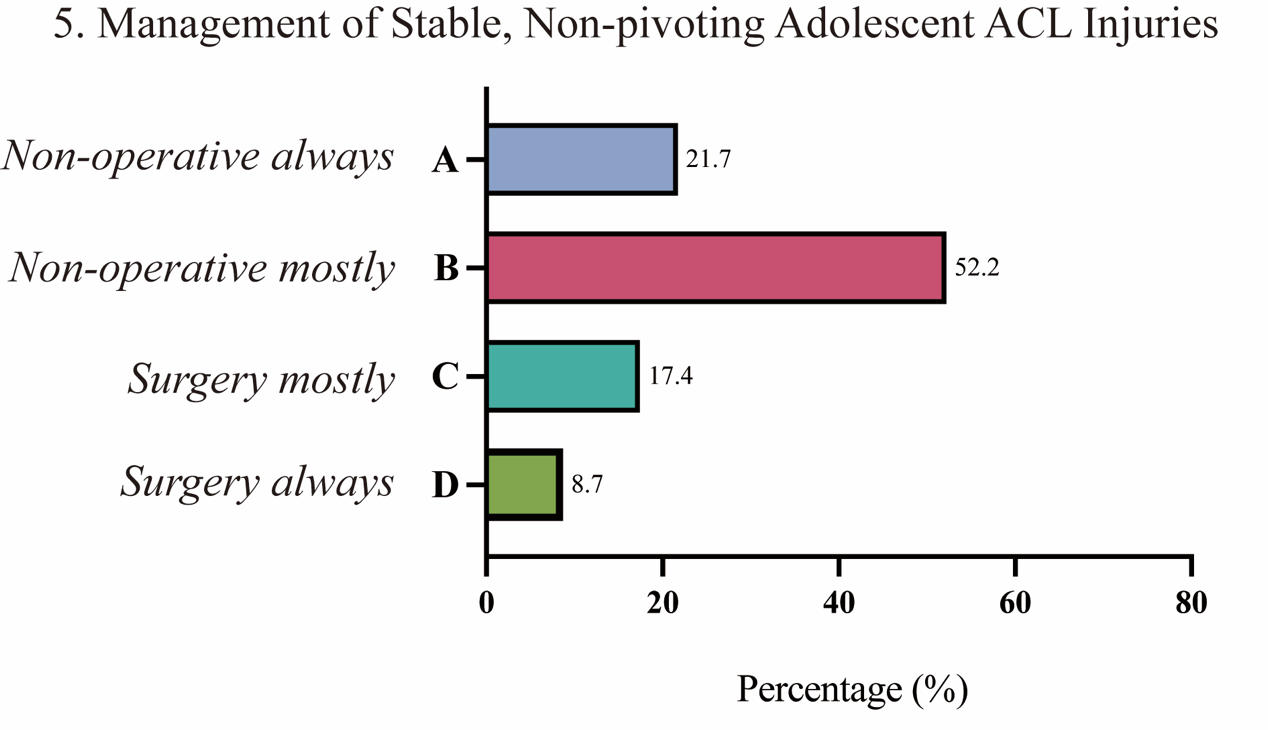
***

***6. What is your typical first-line management approach for paediatric patients with ACL injuries, considering the risk of secondary chondral and meniscal injuries?***

*□ A. I always start with activity modification, bracing, and closed-chain rehabilitation*

*□ B. I usually start with conservative measures but may recommend early surgery in some cases*

*□ C. I often recommend early surgical intervention to prevent secondary injuries*

***
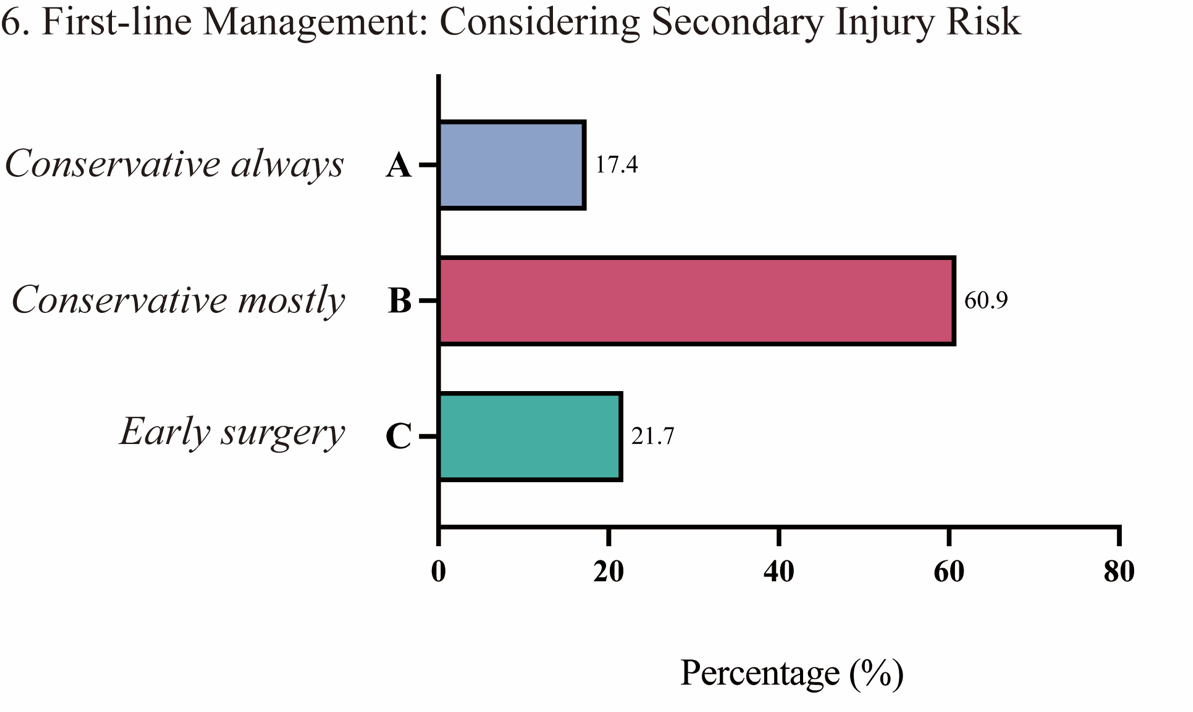
****□ D. Other (please specify): _______________*

***7. Which of the following factors most strongly influence your decision to recommend surgical management for ACL tears in skeletally immature patients? (Select all that apply)***

*□ A. Persistent instability following rehabilitation*

*□ B. Concomitant meniscus or chondral injuries*

*□ C. Impaired knee function or range of motion*

*□ D. Patient's desire to return to competitive pivoting sports*

*□ E. Patient/family preference*


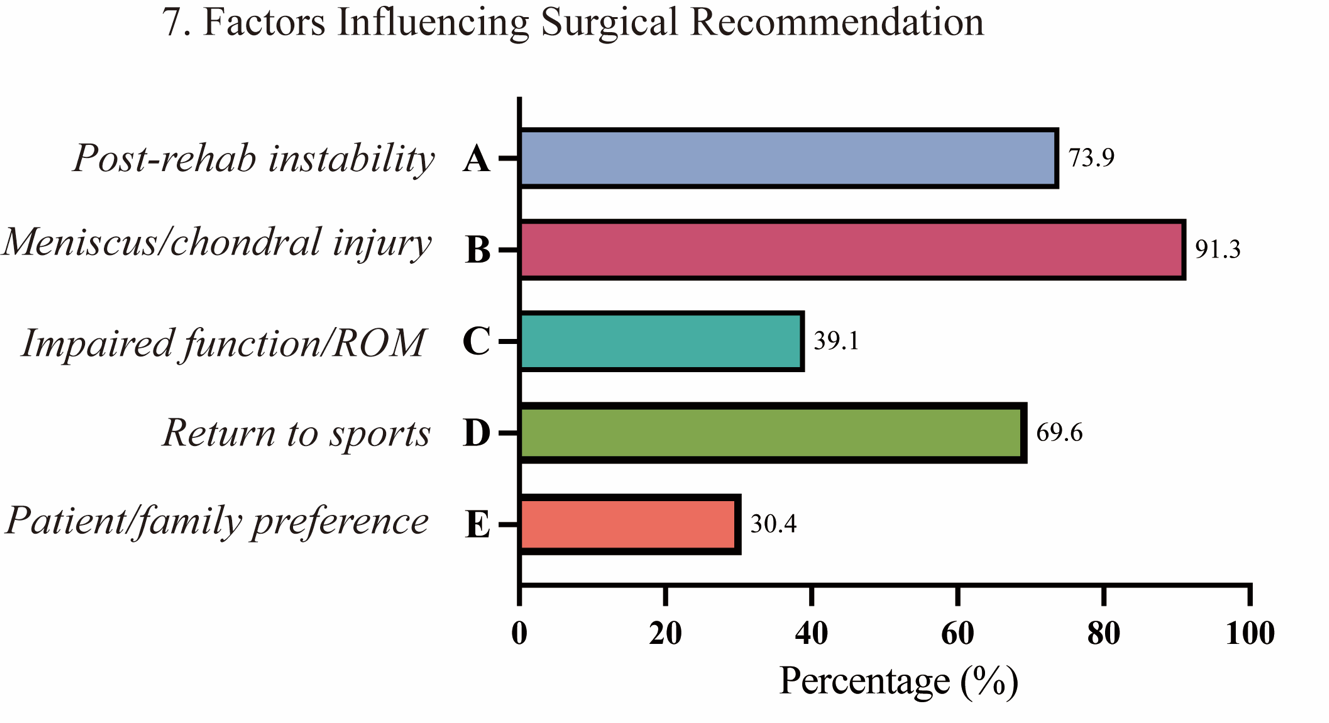
*□ F. Other (please specify): _______________*

***8. How do you primarily assess skeletal maturity when treating ACL injuries in children? (Check all that apply)***

*□ A. Chronological age*

*□ B. Physiologic age (e.g., Tanner staging)*

*□ C. Skeletal age (e.g., Greulich and Pyle Method)*

*□ D. MRI*

*□ E. Other (please specify): _______________*

***
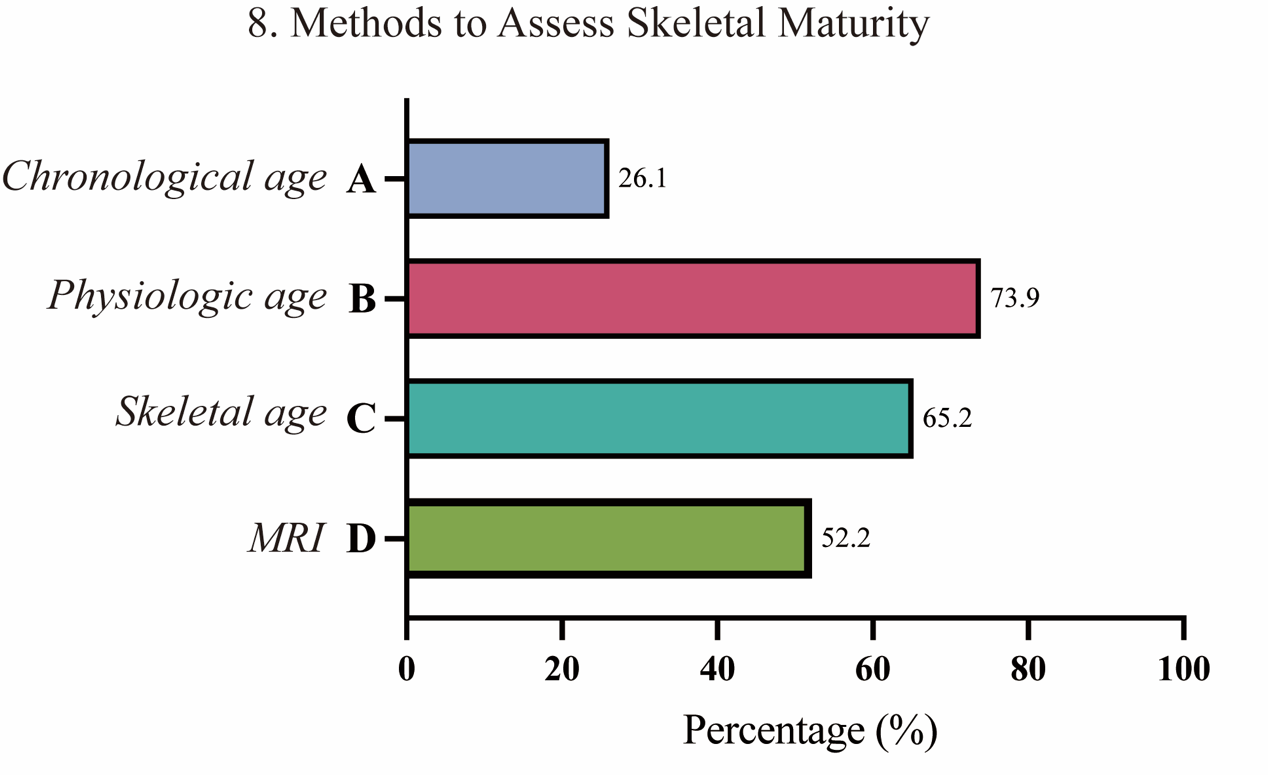
***

***9. Which ACL reconstruction technique is recommended for a child with a bone age of 12 years or less in a male and 11 years or less in a female and Tanner stage 1 or 2?***

*□ A. Physeal-sparing ACL reconstruction using an over-the-top technique*

*□ B. Physeal-sparing ACL reconstruction using all-epiphyseal technique*

*□ C. Partial transphyseal ACL reconstruction*

*□ D. Transphyseal ACL reconstruction*

*□ E. Other (please specify): _______________*

***
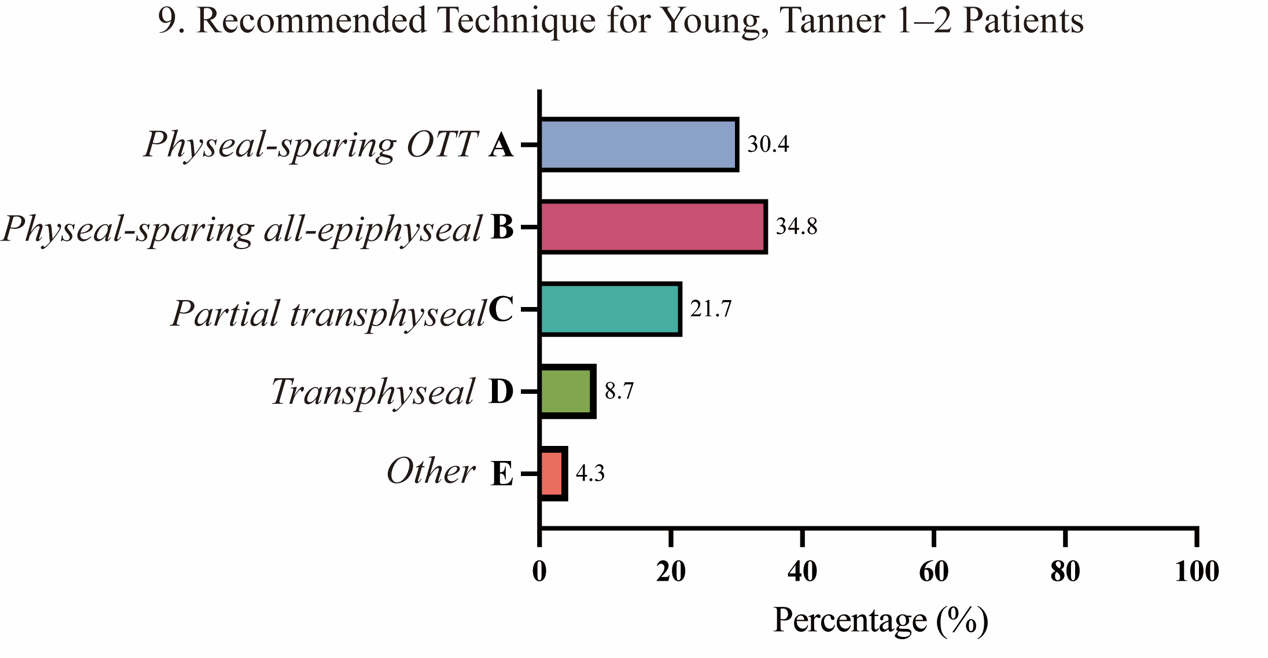
***

***10.*** ***Which ACL reconstruction technique is recommended for an adolescent with a bone age of 13–15 in a male or 12–13 in a female and Tanner stage 3 or greater?***

*□ A. Physeal-sparing ACL reconstruction using an over-the-top technique*

*□ B. Physeal-sparing ACL reconstruction using all-epiphyseal technique*

*□ C. Partial transphyseal ACL reconstruction*

*□ D. Transphyseal ACL reconstruction*

*□ E. Other (please specify): _______________*

***
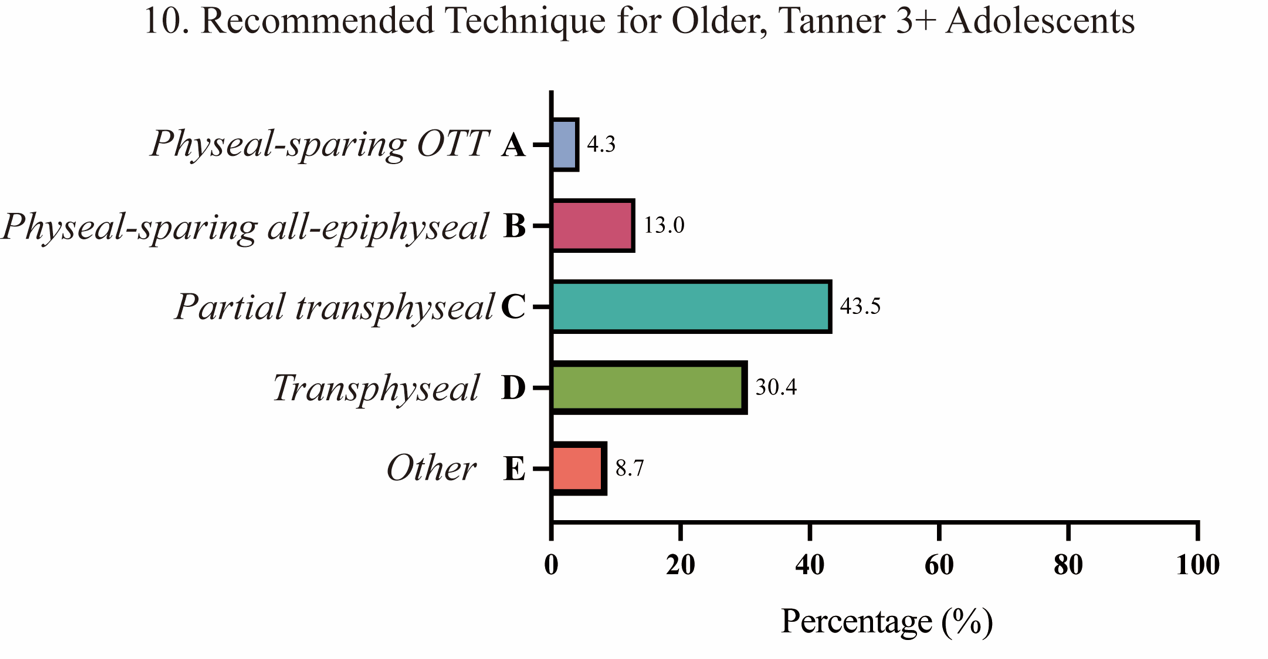
***

***11. Which ACL reconstruction technique is recommended for an older adolescent with closing physes, with a bone age of 16 or older in a male and 14 or older in a female and Tanner stage 4 or 5?***

*□ A. Physeal-sparing ACL reconstruction using an over-the-top technique*

*□ B. Physeal-sparing ACL reconstruction using all-epiphyseal technique*

*□ C. Partial transphyseal ACL reconstruction*

*□ D. Transphyseal ACL reconstruction*

*□ E. Other (please specify): _______________*

***
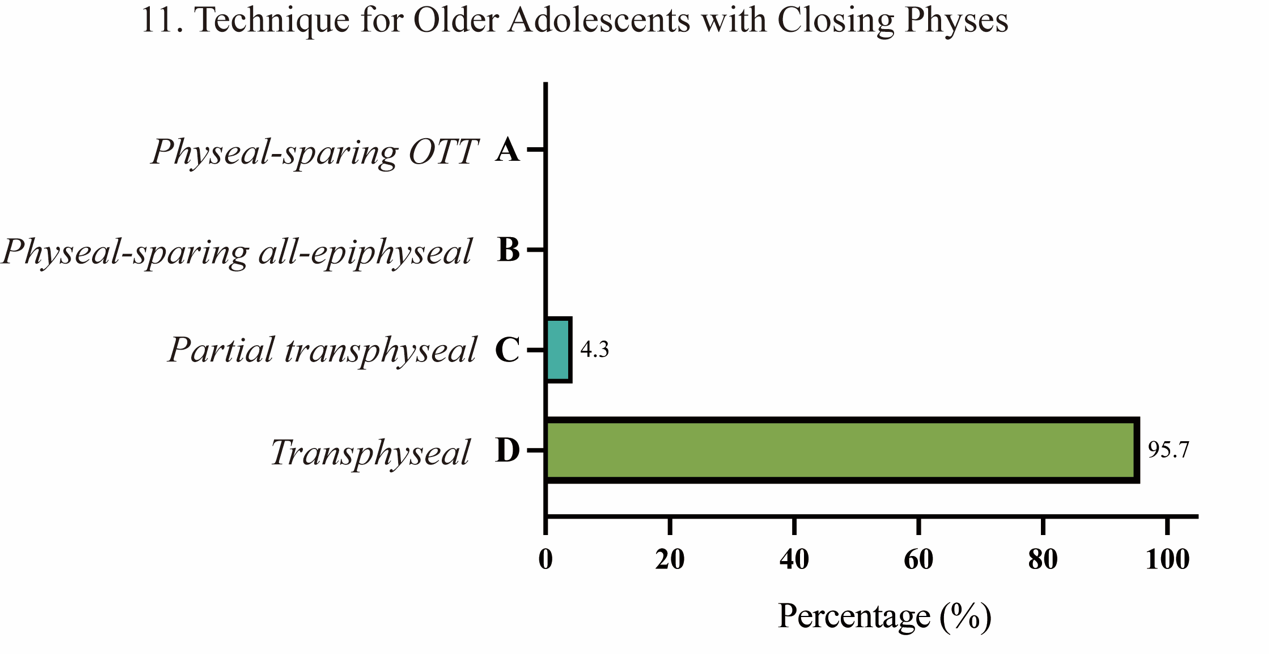
***

***Surgical Timing***

***12. When do you typically perform ACL reconstruction in paediatric patients?***

*□ A. As soon as possible (within 3 weeks of injury)*

*□ B. After a short delay (3-6 weeks)*

*□ C. After extended rehabilitation (6-12 weeks)*

*□ D. Delay surgery until skeletal maturity*

*□ E. Other (please specify): _______________*

***
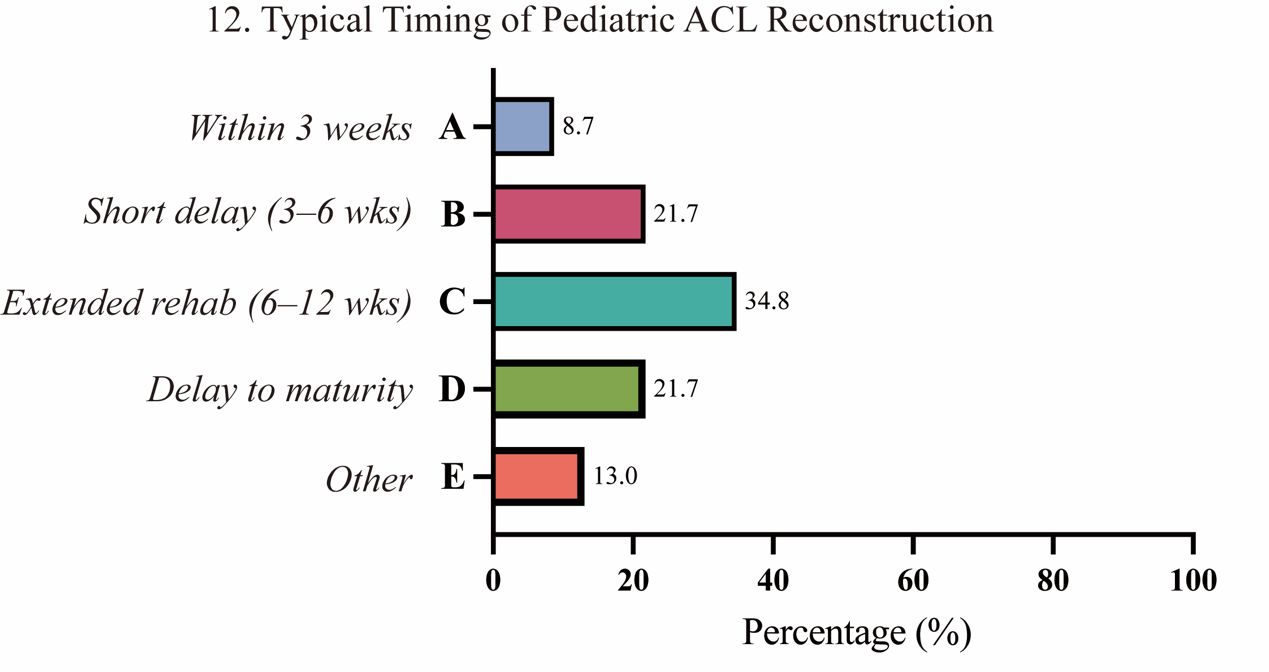
***

***13. How do you typically approach cases of unstable meniscal tears with ACL injury in adolescent patients?***

*□ A. Prioritize meniscal repair and delay ACL reconstruction until skeletal maturity*

*□ B. I typically perform meniscal repair and ACL reconstruction simultaneously*

*□ C. Other (please specify): _______________*

***
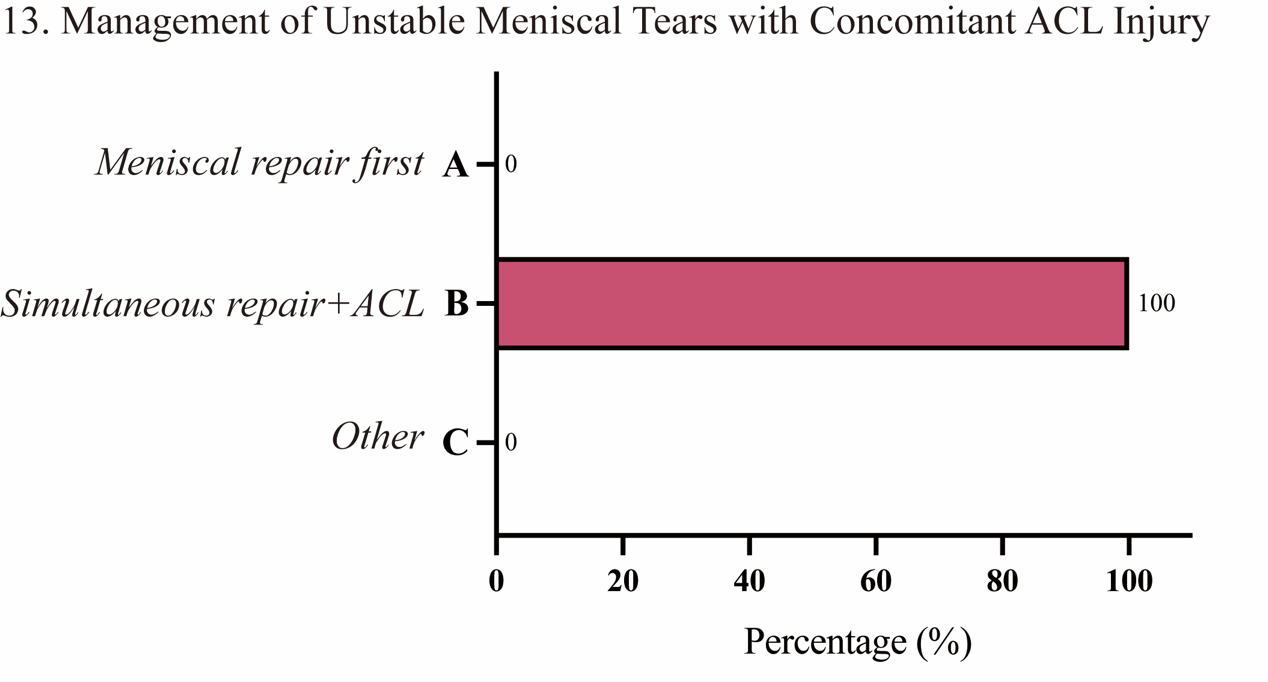
***

***14. How do you approach the use of lateral extra-articular tenodesis (LET) in adolescent ACL reconstruction?***

*□ A. I routinely perform LET with ACL reconstruction in adolescents*

*□ B. I selectively use LET in high-risk cases (e.g., revision surgery, high-level athletes)*

*□ C. I rarely use LET in adolescent ACL reconstruction*

*□ D. I never use LET in adolescent patients*

*□ E. Other (please specify): _______________*

***
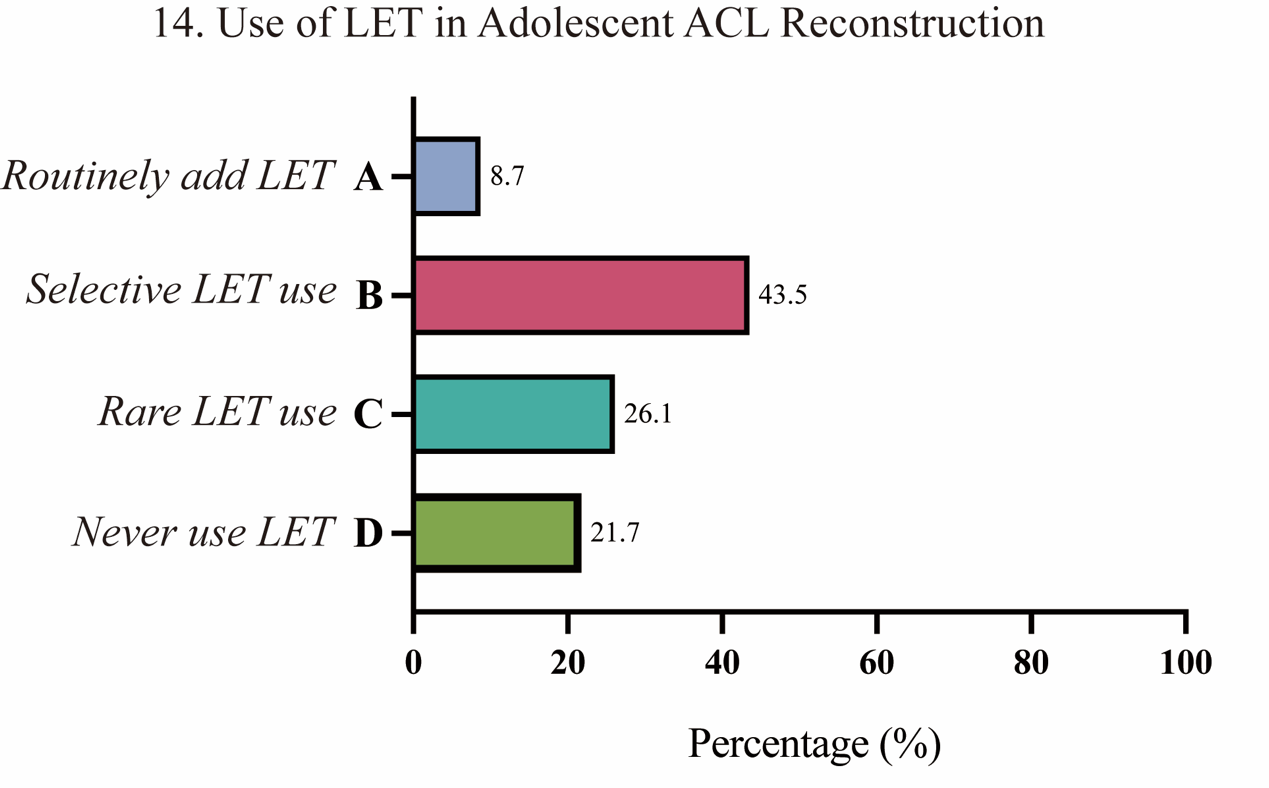
***

***Section 4: Graft Choice and surgical techniques***

***15. What is your preferred graft choice for all-epiphyseal ACL reconstruction in adolescents?***

*□ A. Hamstring autograft*

*□ B. Quadriceps tendon autograft*

*□ C. Bone-patellar tendon-bone (BTB) autograft*

*□ D. Allograft*

*□ E. It depends on individual patient factors*

*□ F. Other (please specify): _______________*

***
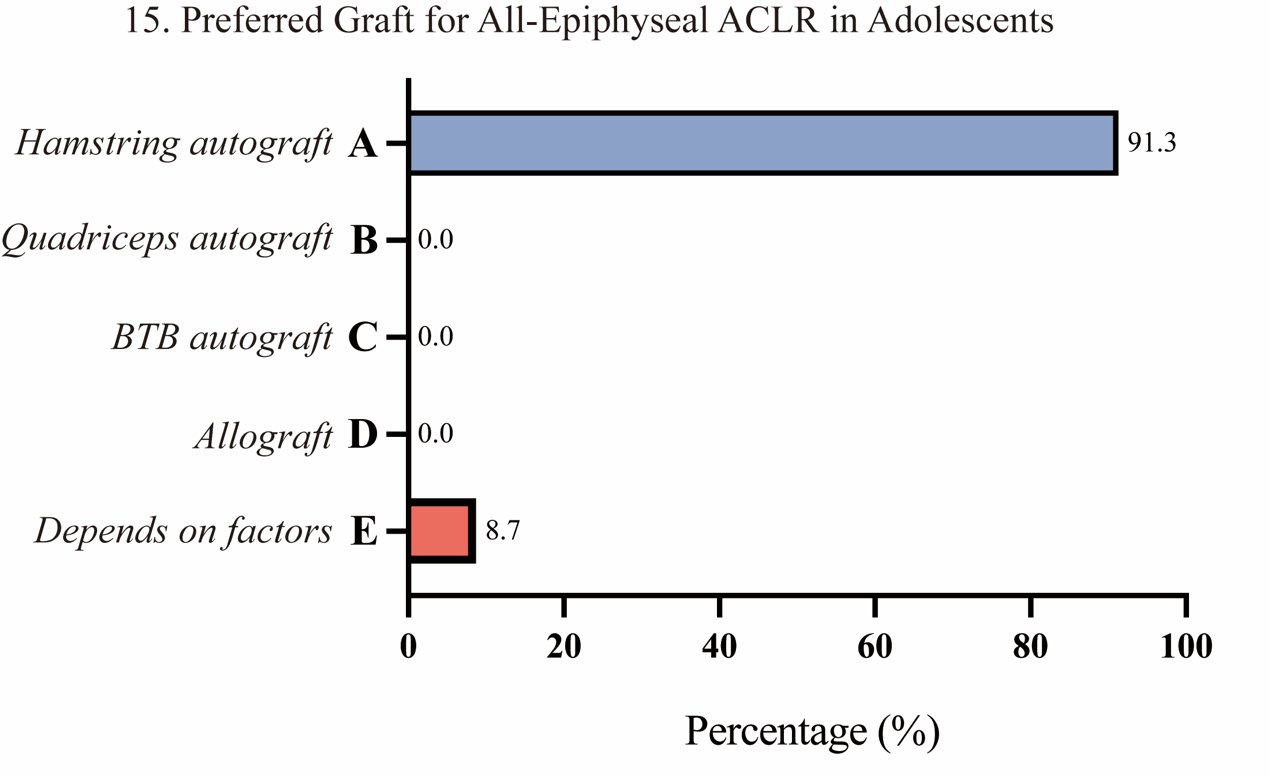
***

***16. How do you approach using bone-patellar tendon-bone (BTB) autografts in paediatric patients with open physes?***

*□ A. I routinely use BTB autografts regardless of skeletal maturity*

*□ B. I use BTB autografts only in patients nearing skeletal maturity*

*□ C. I avoid BTB autografts in all patients with open physes*

*□ D. I don't use BTB autografts in my practice*

*□ E. Other (please specify): _______________*

***
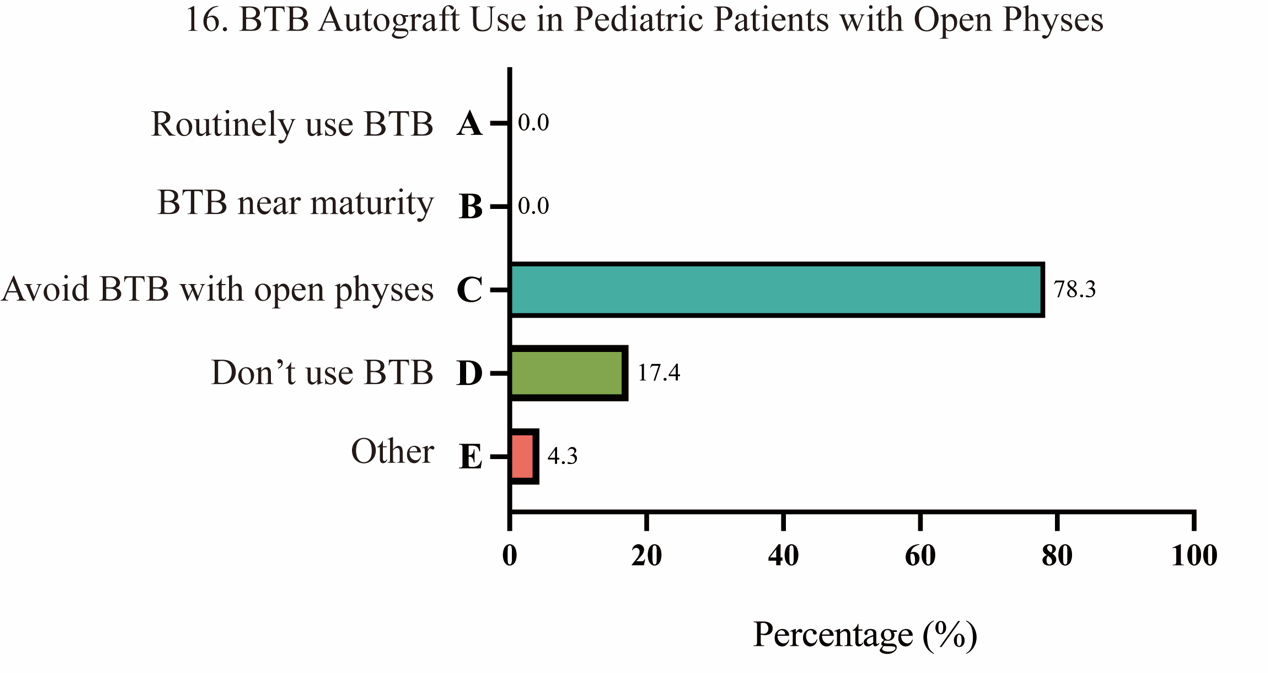
***

***17. How do you approach the use of allografts for ACL reconstruction in adolescent patients?***

*□ A. I routinely use allografts in adolescents*

*□ B. I use allografts in select cases (e.g., revision surgeries, multi-ligament injuries)*

*□ C. I avoid using allografts in adolescents due to higher failure rates*

*□ D. I don't use allografts in my practice*

*□ E. Other (please specify): _______________*

***
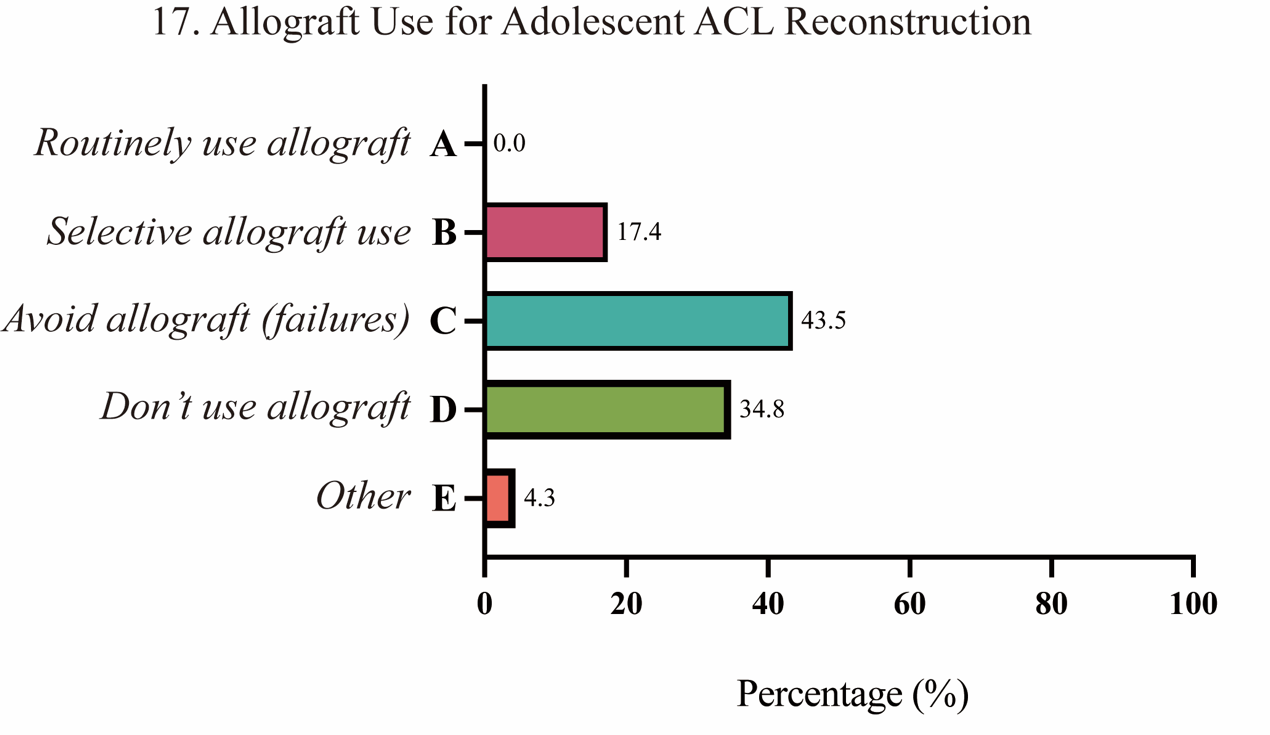
***

***18.*** ***The optimal graft diameter for ACL reconstruction in paediatric patients has yet to be definitively established.*** ***What graft diameter do you typically aim for in transphyseal ACL reconstruction for adolescent patients?***

*□ A. <6 mm*

*□ B. 6-7 mm*

*□ C. 7-8 mm*

*□ D. Greater than 8 mm*

*□ E. I don't have a specific target diameter; it varies by patient*

*□ F. Other (please specify): _______________*

*
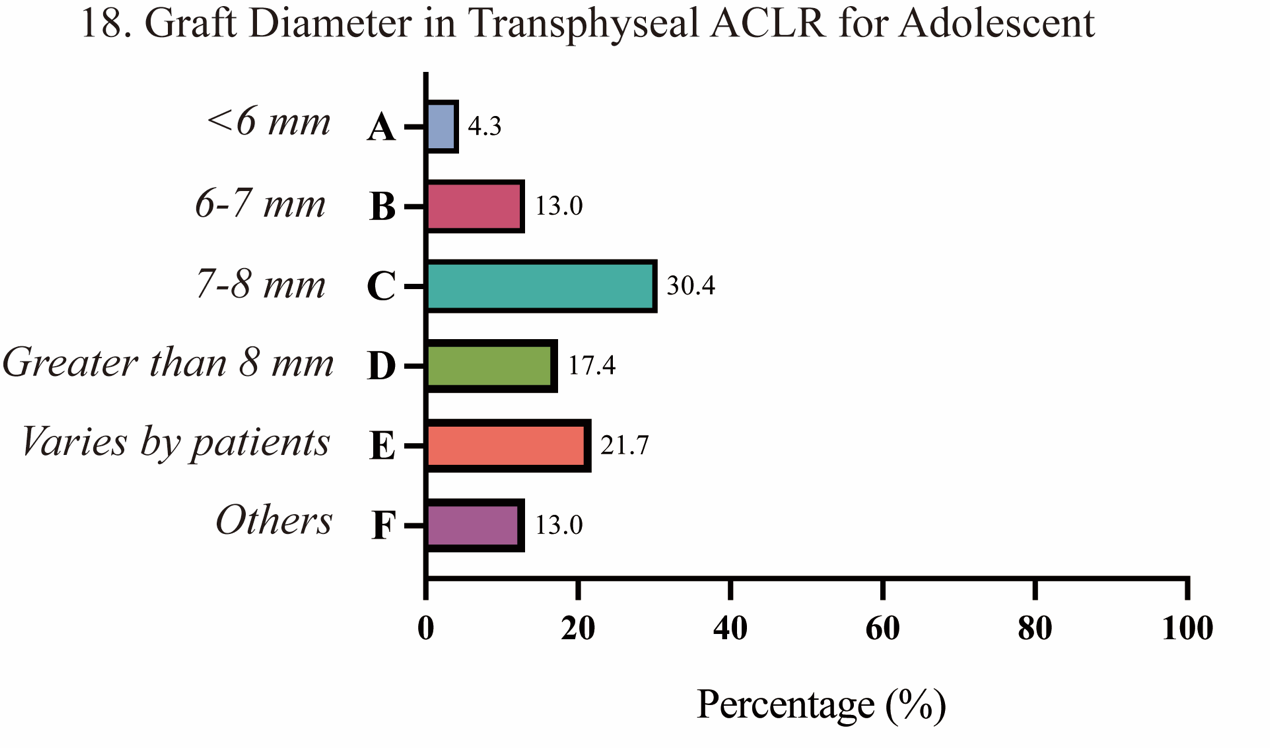
*

***19. Which ACL reconstruction technique do you typically use for skeletally immature patients?***

*□ A. I always perform single-bundle reconstruction*

*□ B. I usually perform single-bundle reconstruction but consider double-bundle in some cases*

*□ C. I usually perform double-bundle reconstruction but use single-bundle in some cases*

*□ E. I always perform double-bundle reconstruction*

*□ F. I don't perform ACL reconstructions in skeletally immature patients*

*□ G. Other (please specify): _______________*

***
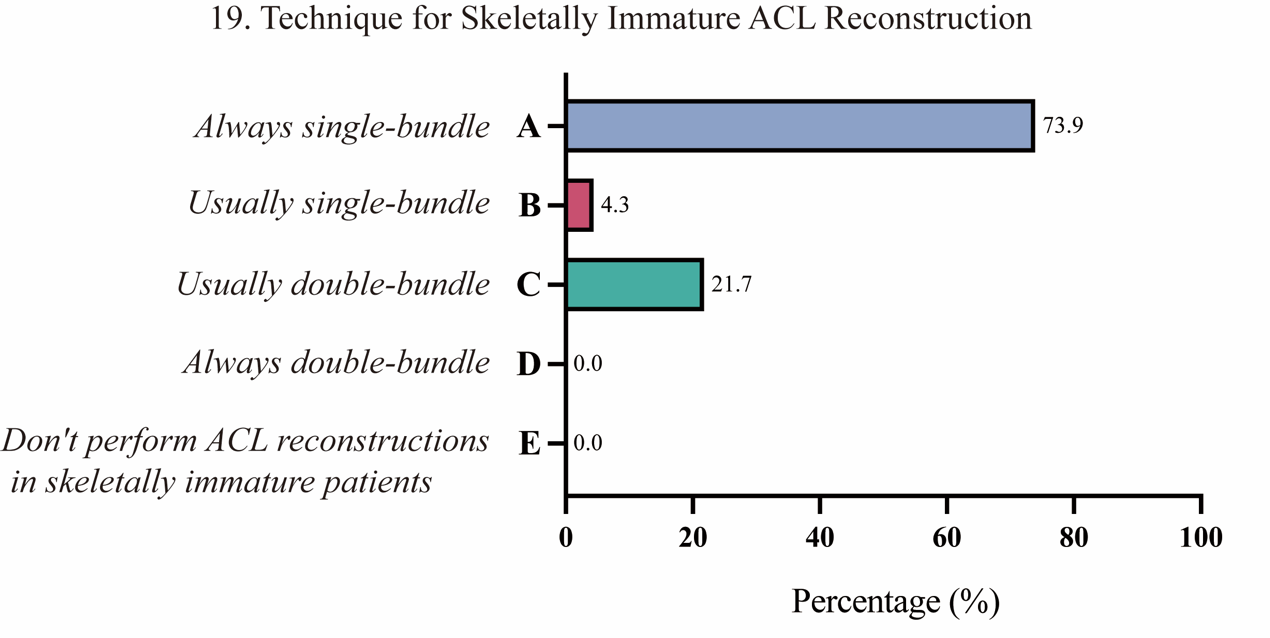
***

***Section 5: Postoperative Care and Risk Management***

***20. How do you approach postoperative monitoring for growth disturbances (e.g., leg length discrepancy or angular deformity) in paediatric patients following ACL reconstruction?***

*□ A. I routinely monitor all paediatric patients, regardless of skeletal maturity*

*□ B. I routinely monitor patients with significant growth remaining*

*□ C. I monitor selectively based on individual risk factors*

*□ D. I only monitor if clinical symptoms or concerns arise*

*□ E. I perform more frequent and intensive monitoring for all paediatric ACL reconstruction patients*

*□ F. Other (please specify): _______________*

*
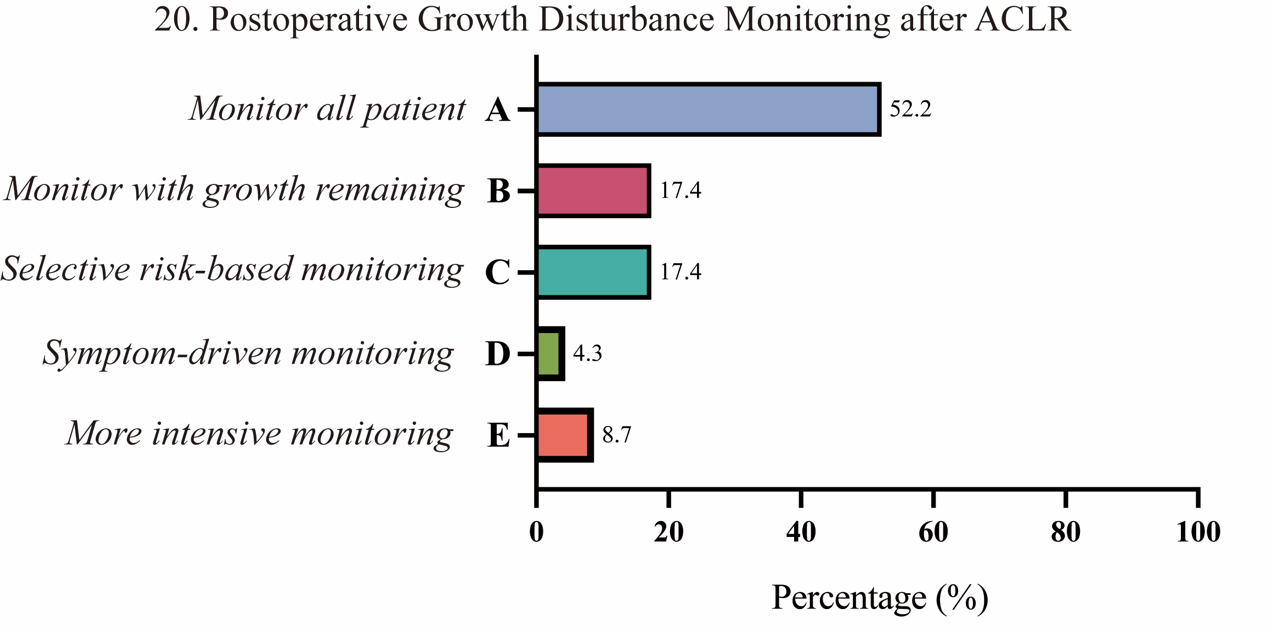
*

***Section 6: Associated Injuries and Complications***

***21. How do you manage concomitant meniscal tears in paediatric ACL injuries?***

*□ A. I attempt meniscal repair whenever possible, regardless of the tear pattern*

*□ B. I selectively repair based on tear pattern and location (zones)*

*□ C. I primarily perform partial meniscectomy*

*□ D. I initially attempt non-operative management for most meniscal tears*

*□ E. Other (please specify): _______________*

*
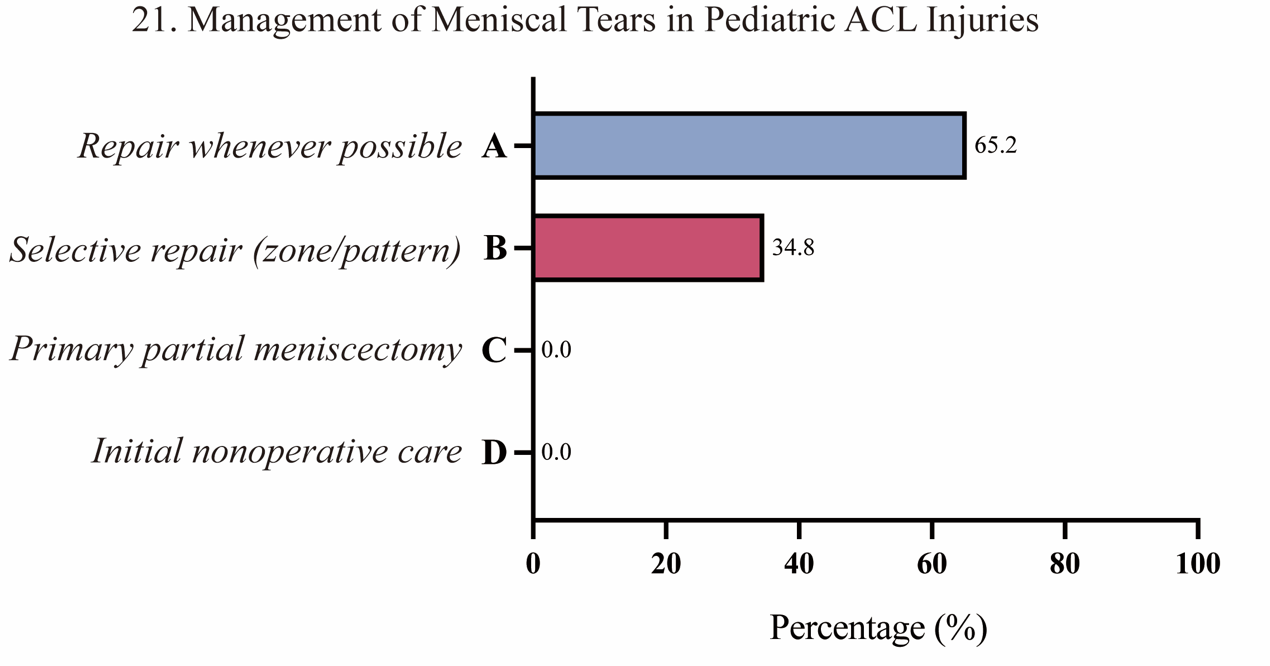
*

***22. What strategies do you primarily employ in your practice to prevent contralateral ACL injury in paediatric patients after ACL reconstruction? (Select all that apply)***

*□ A. Implement bilateral rehabilitation protocols*

*□ B. Recommend delayed return to sport*

*□ C. Prescribe specific neuromuscular training programs*

*□ D. Advise long-term activity modification*

*□ E. Use functional testing to guide return-to-sport decisions*

*□ F. Recommend continued use of protective bracing*

*□ G. Provide education on proper movement patterns and technique*

*□ H. Suggest regular follow-up assessments even after return to sport*

*□ I. Other (please specify): _______________*

***
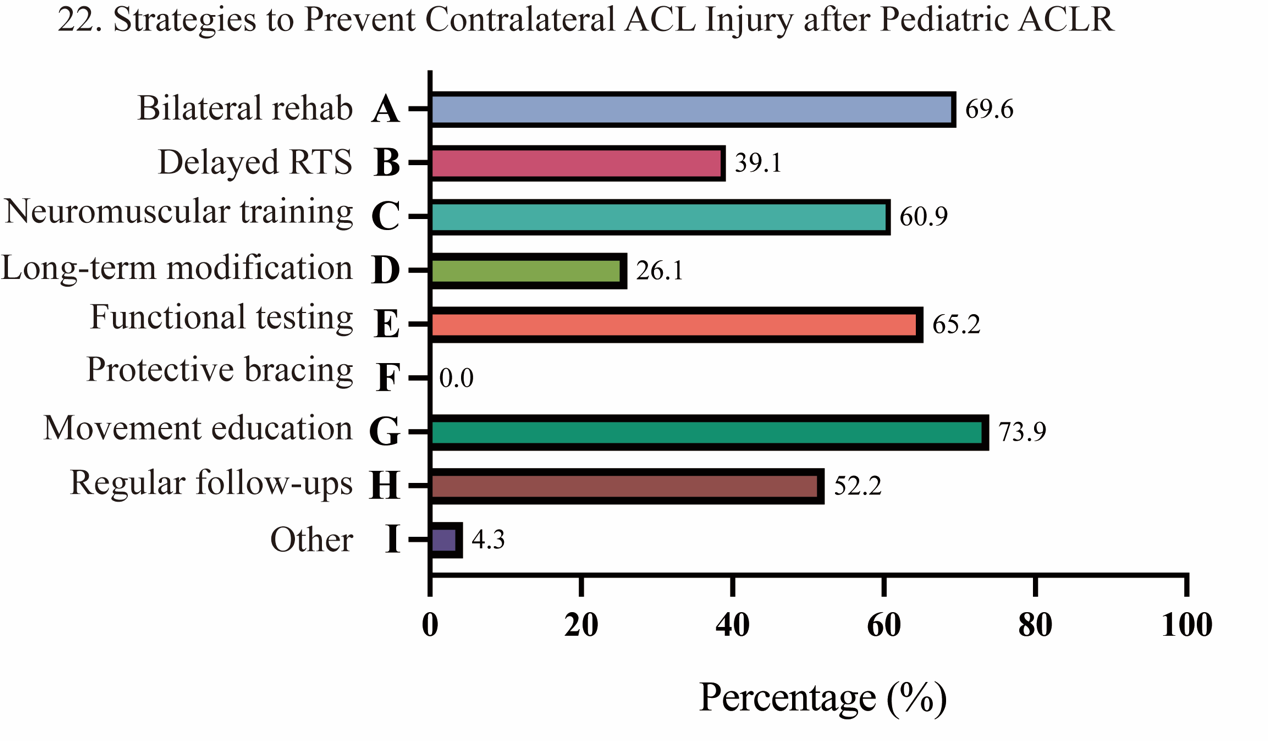
***

***Section 7: Rehabilitation and return to sport***

***23. How do you approach your practice's ACL rehabilitation protocols for paediatric patients?***

*□ A. I use paediatric-specific protocols tailored to children*

*□ B. I use modified adult protocols with some adjustments for paediatric patients*

*□ C. I use standard adult protocols with minimal modifications*

*□ D. I don't use set protocols; I develop individualized plans for each patient*

*□ E. I refer all my paediatric ACL patients to specialized paediatric physical therapists*

*□ Other (please specify): _______________*

*
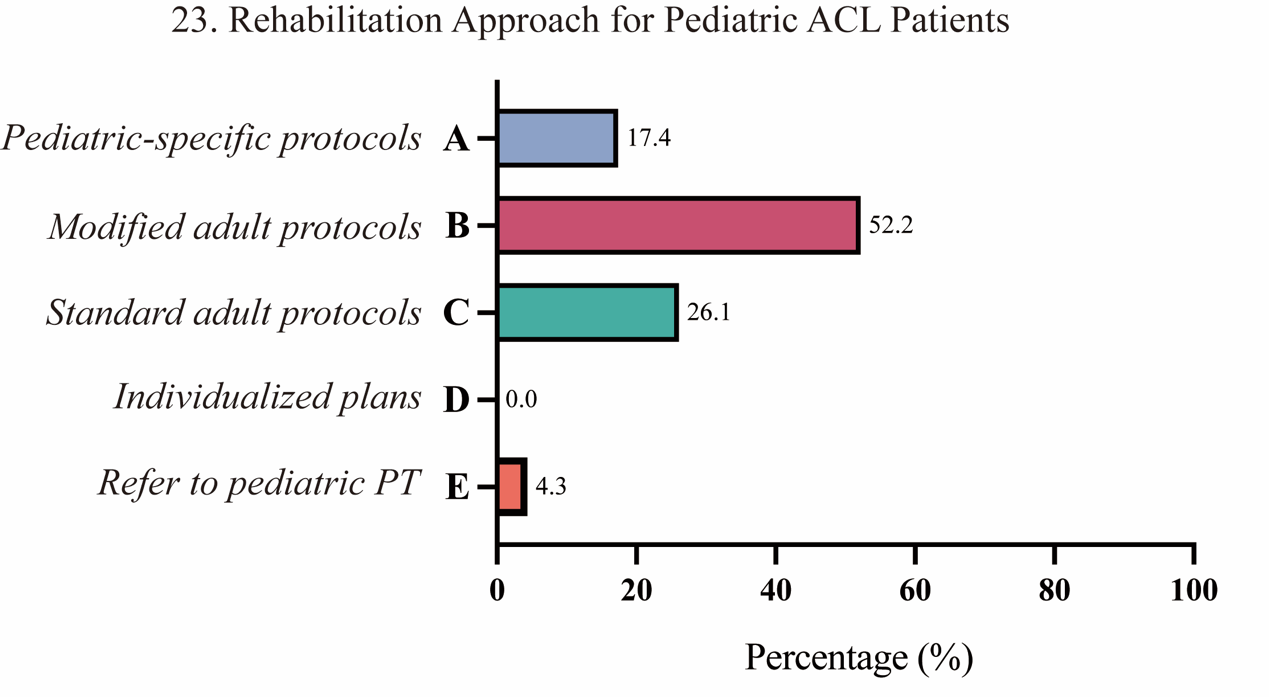
*

***24. What are your primary criteria for allowing a return to sports after paediatric ACL reconstruction? (Select all that apply)***

*□ A. Time from surgery (please specify typical timeframe: ___ months)*

*□ B. Strength testing (e.g., quadriceps/hamstring strength)*

*□ C. Functional testing (e.g., single-leg hop tests)*

*□ D. Range of motion assessment*

*□ E. Neuromuscular control evaluation*

*□ F. Psychological readiness assessment*

*□ G. Sport-specific skills assessment*

*□ H. Combination of objective measures and subjective clinical judgment*

*□ I. Patient/family education and shared decision-making*

*□ J. Other (please specify): _______________*

*
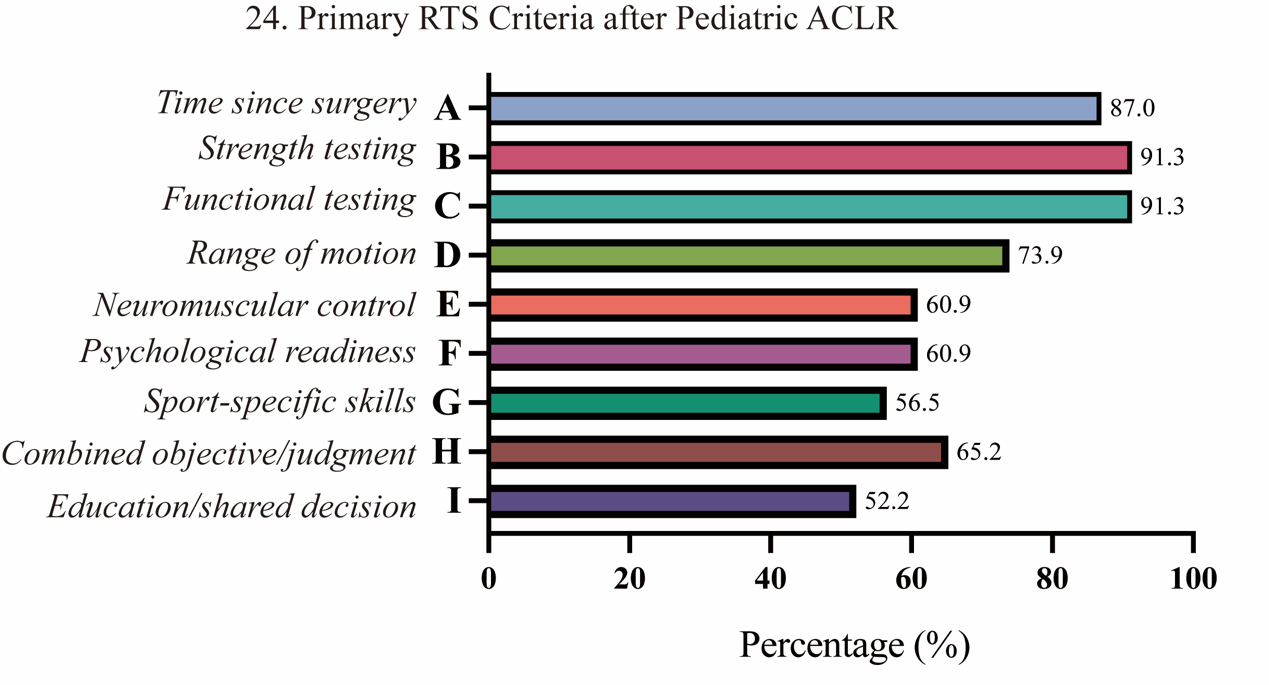
*

***Section 8: Outcomes evaluation***

***25. Which outcome measures do you routinely use to evaluate paediatric ACL reconstruction results? (Select all that apply)***

*□ A. Return to sport rate*

*□ B. Growth disturbance incidence*

*□ C. Patient-reported outcome measures (PROMs)*

*□ D. Re-injury rate*

*□ E. Functional testing results (e.g., hop tests, agility drills)*

*□ F. Strength testing results*

*□ G. Range of motion measurements*

*□ H. Knee laxity measurements (e.g., KT-1000)*

*□ I. Patient/parent satisfaction*

*□ J. Other (please specify): _______________*

*
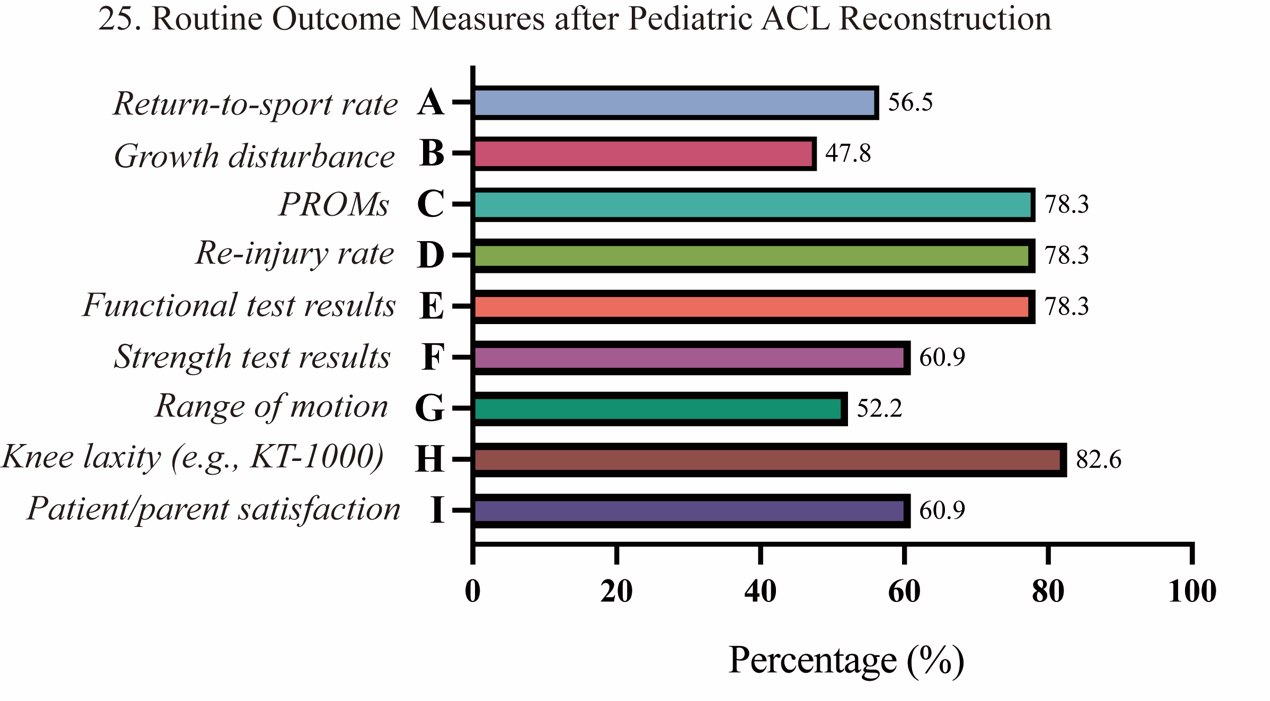
*

***26. Regarding Patient-Reported Outcome Measures (PROMs) for paediatric ACL patients, which statement best describes your current practice?***

*□ A. I use paediatric-specific PROMs designed for ACL patients*

*□ B. I use general paediatric orthopaedic PROMs*

*□ C. I use adult ACL PROMs, modified for paediatric patients*

*□ D. I use standard adult ACL PROMs without modification*

*□ E. I don't routinely use PROMs in my paediatric ACL patients*

*□ F. Other (please specify): ___________*

*
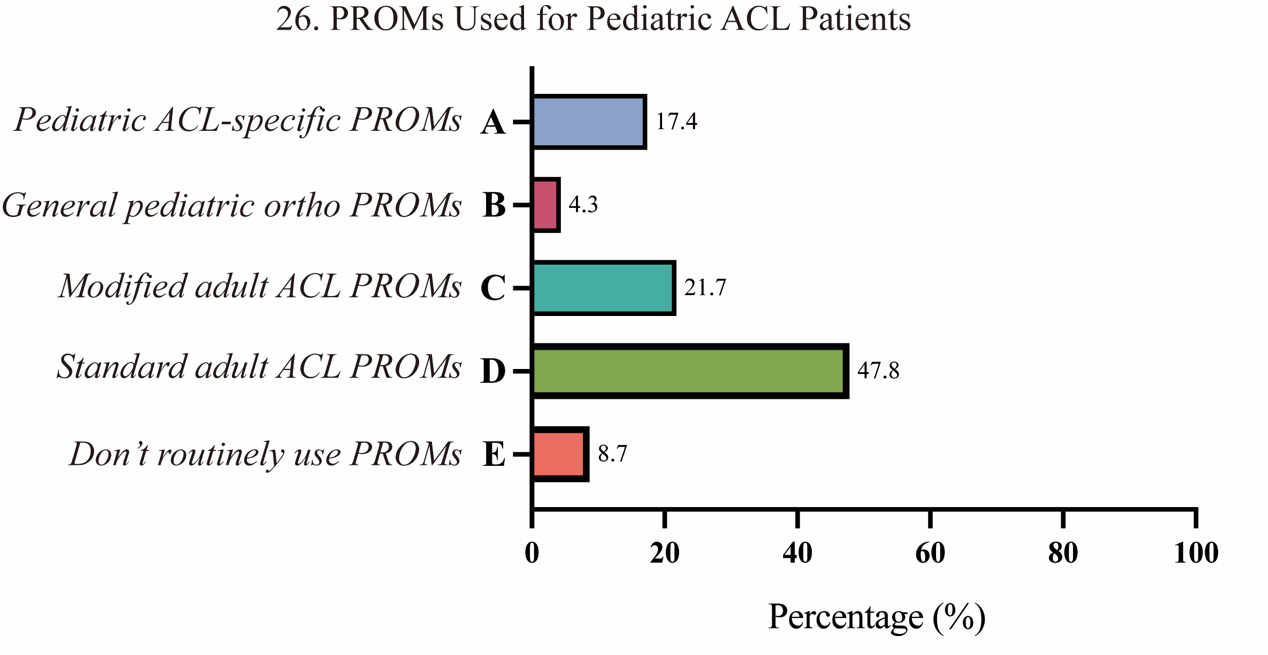
*

***Section 9. Future Directions***

***27. What are the most critical areas for future research in paediatric ACL injuries? (Select up to three)***

*□ A. Prevention strategies*

*□ B. Diagnostic techniques*

*□ C. Surgical techniques*

*□ D. Rehabilitation protocols*

*□ E. Growth disturbance prevention and management*

*□ F. Psychological aspects and return-to-sport readiness*

*□ G. Paediatric-specific outcome measures*

*□ H. Biological augmentation (e.g., growth factors, stem cells)*

*□ I. Other (please specify): _______________*

***
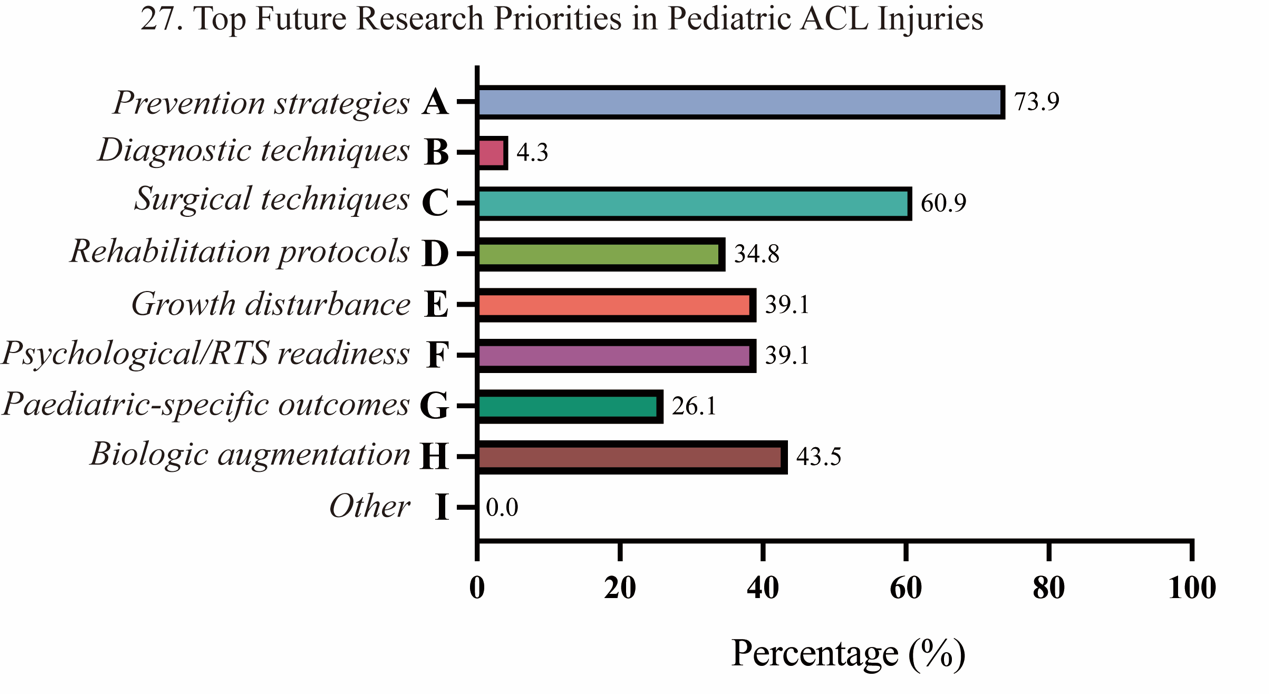
***

***28. Regarding current guidelines for managing paediatric ACL injuries, which statement best reflects your view?***

*□ A. Current guidelines are sufficient and do not need significant changes*

*□ B. Current guidelines are good but need minor updates or refinements*

*□ C. More comprehensive, evidence-based guidelines are needed*

*□ D. Other (please specify): _______________*


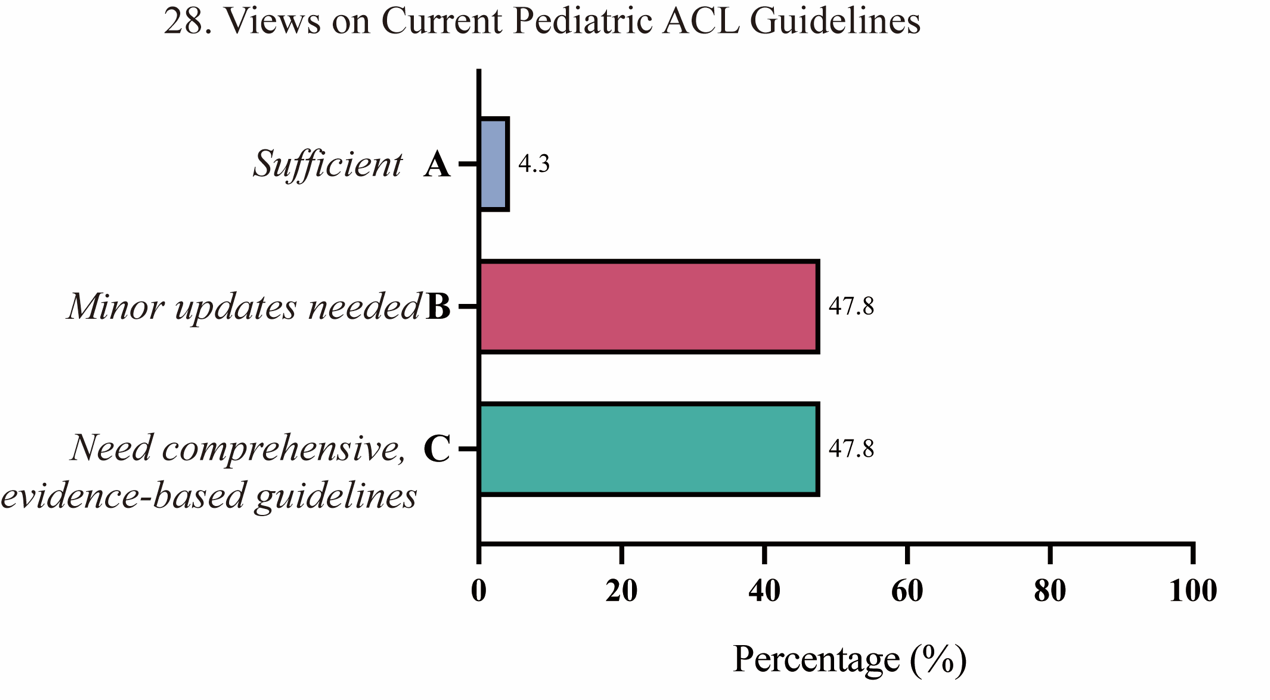

Supplement: Multimedia component 1 [file mmc1.docx]
